# Widespread occurrence of pesticides in low-income housing

**DOI:** 10.1038/s41370-024-00665-y

**Published:** 2024-06-22

**Authors:** Sara Vaezafshar, Jeffrey A. Siegel, Liisa Jantunen, Miriam L. Diamond

**Affiliations:** 1 Department of Earth Sciences, University of Toronto, Toronto, ON M5S 3B1 Canada; 2 Department of Civil and Mineral Engineering, University of Toronto, Toronto, ON M5S 1A4 Canada; 3 Dalla Lana School of Public Health, University of Toronto, Toronto, ON M5T 3M7 Canada; 4 Air Quality Processes Research, Environment and Climate Change Canada, Egbert, ON L0L 1N0 Canada; 5 School of Environment, University of Toronto, Toronto, ON M5S 3E8 Canada

**Keywords:** Indoor current-use pesticide air concentrations, Indoor legacy pesticide air concentrations, Indoor air quality, Low socioeconomic status, Social housing, Tobacco-related pesticides

## Abstract

**Background:**

Low socioeconomic status (SES) residents living in social housing, which is subsidized by government or government-funded agencies, may have higher exposures to pesticides used in indoor residences since pesticides are applied due to structural deficiencies, poor maintenance, etc.

**Objective:**

To estimate exposure of residents in low-SES social housing built in the 1970s to legacy and current-use pesticides and to investigate factors related to exposures.

**Methods:**

Twenty-eight particle-phase pesticides were measured in the indoor air of 46 units in seven low-income social housing, multi-unit residential buildings (MURBs) in Toronto, Canada using portable air cleaners deployed for 1 week in 2017. Pesticides analyzed were legacy and current use in the classes: organochlorines, organophosphates, pyrethroids, and strobilurins.

**Results:**

At least one pesticide was detected in 89% of the units with detection frequencies (DF) for individual pesticides of up to 50%, including legacy organochlorines and current-use pesticides. Current-use pyrethroids had the highest DF and concentrations, with the highest particle-phase concentration for pyrethrin I at 32,000 pg/m^3^. Heptachlor, restricted for use in Canada in 1985, had the highest estimated maximum total air (particle plus gas phase) concentration of 443,000 pg/m^3^. Heptachlor, lindane, endosulfan I, chlorothalonil, allethrin, and permethrin (except in one study) had higher concentrations than those measured in low-income residences reported elsewhere. In addition to the intentional use of pesticides to control pests and their use in building materials and paints, tobacco smoking was significantly correlated with the concentrations of five pesticides used on tobacco crops. The distribution of pesticides with high DF in individual buildings suggested that pest eradication programs by the building management and/or pesticide use by residents were the major sources of measured pesticides.

**Impact:**

Low-income social housing fills a much-needed demand, but the residences are prone to pest infestation and hence pesticide use. We found exposure to at least 1 of 28 particle-phase pesticides in 89% of all 46 units tested, with the highest DF and concentrations for current-use pyrethroids and long-banned organochlorines (e.g., DDT, heptachlor) due to very high persistence indoors. Also measured were several pesticides not registered for use indoors, e.g., strobilurins used to treat building materials and pesticides used on tobacco crops. These results, which are the first Canadian data for most pesticides indoors, show widespread exposure to numerous pesticides.

## Introduction

Pesticides are widely used on agricultural crops to minimize losses due to pests. In 2018, about 72% of pesticides sold in Canada were used in agriculture compared with only 4.5% in residential settings [1]. It is logical then, that most research on pesticide concentrations and exposures has focused on agricultural situations [2–4], leaving many gaps regarding pesticide profiles and levels in homes where they are also used typically to control pests. A single application indoors in a residential setting can result in the introduction of 15 mg of pesticides [5]. Pesticides are used indoors to control vermin such as cockroaches and bed bugs. Additional uses of pesticides are to control pests on pets and as biocidal treatments in furnishings and consumer products (e.g., woolen carpets, textiles), and construction materials (e.g., fungicide-containing wall paint, mold-resistant drywall) [6–9]. Further, activities of residents, such as indoor smoking, can introduce pesticides applied to tobacco crops [10]. Transport from outside to indoors is another source of pesticides indoors [11–13].

Outside of farm workers and their families, certain populations are vulnerable to pesticide exposure. Children are more likely than adults to be exposed to many indoor contaminants, including pesticides, because of their higher rates of inhalation relative to body mass, dust ingestion, and hand-to-mouth behavior [14, 15]. For example, Trunnelle et al. found a positive correlation between pyrethroid/pyrethrin (PYRs) concentrations in floor wipes and the concentration of PYR metabolites in children’s urine [16]. DF reported by the Canadian Health Measurement Survey (CHMS) for PYR pesticide metabolites were higher in children ages 3–5 years than in older age classes [17]. Pregnant women and their fetuses are also considered as a vulnerable group due to the risk of early-life exposure to pesticides. Whyatt et al. reported that pesticides in mothers’ and newborns’ blood samples were highly correlated, consistent with maternal transfer to the fetus [18].

Individuals living in low-quality or low-income housing are at increased risk of exposure to indoor contaminants, including pesticides [19–21]. For example, in Canada, individuals of low socioeconomic status (SES) have been found to experience higher exposures than higher SES individuals to phthalates, halogenated flame retardants, organophosphate plasticizers and flame retardants, and polycyclic aromatic hydrocarbons (PAHs) [22–24]. Some of these findings pertain to individuals living in “social housing”, which we define as rental units subsidized by government (or government-funded agency), intended for low-SES residents [25]. Multi-unit residential building (MURB) social housing is prone to pest infestation primarily due to their structural deficiencies (e.g., cracks and crevices in walls) and the lack of adequate maintenance/repairs, inadequate cleaning services and waste management, and frequent overcrowding [20, 26]. Although integrated pest management programs have been devised to minimize the need for pest eradication programs by building management, which can reduce the risk of exposure to pesticides, especially in multi-family buildings, pests may spread throughout a building [21, 27, 28]. The spread of pests and the attendant use of pesticides will negatively affect indoor air quality and could put residents at risk of pesticide exposure with attendant adverse health outcomes [29]. Several studies conducted in the United States have found exposures to higher levels of banned and current-use pesticides in low- vs high-income housing due to poor housing quality [11, 26, 30–32]. Since low-income residents often have limited means of leaving their homes, they can be continuously exposed to pesticides inside their homes.

In homes, residents can experience prolonged exposure to elevated pesticide levels given their persistence due to reduced degradation pathways via sunlight, moisture, and microbial degradation [33–35]. Concerns arise due to the reported associations between exposures and adverse health impacts such as neurodevelopmental outcomes, notably reduced verbal IQ in boys, as well as hematological cancer, brain cancer (including childhood cancers), outcomes associated with endocrine disruption, and Alzheimer’s disease [36–41].

The environmental persistence and health risks to human and ecosystem health from certain pesticides have prompted restrictions and prohibitions. In 2004, the Stockholm Convention listed the “dirty dozen” Persistent Organic Pollutants (POPs) for global elimination pertaining to all applications, including nine organochlorine pesticides (OCPs), namely aldrin, chlordane, dichlorodiphenyltrichloroethane (DDT), dieldrin, endrin, heptachlor, hexachlorobenzene, mirex, and toxaphene [42]. Organophosphate pesticides (OPPs) (e.g., chlorpyrifos and ethyl parathion) and carbamate insecticides (e.g., carbaryl and carbofuran) are largely restricted for indoor use in Europe and North America while some outdoor applications still continue [43–47]. In response to these restrictions, PYRs have seen increased use [48]. Human exposure to PYRs is widespread. According to the CHMS, at least three PYRs metabolites were detected in more than 65% of 3000 urine samples from Canadians aged 6–79 [48]. PYRs have been regarded as a class of pesticides with low acute toxicity for mammals, however, recent studies have reported potential health effects associated with exposures such as damage to the reproductive system, and neurobehavioral problems and neurodevelopmental disorders in children [49–53].

**Fig. 1:**
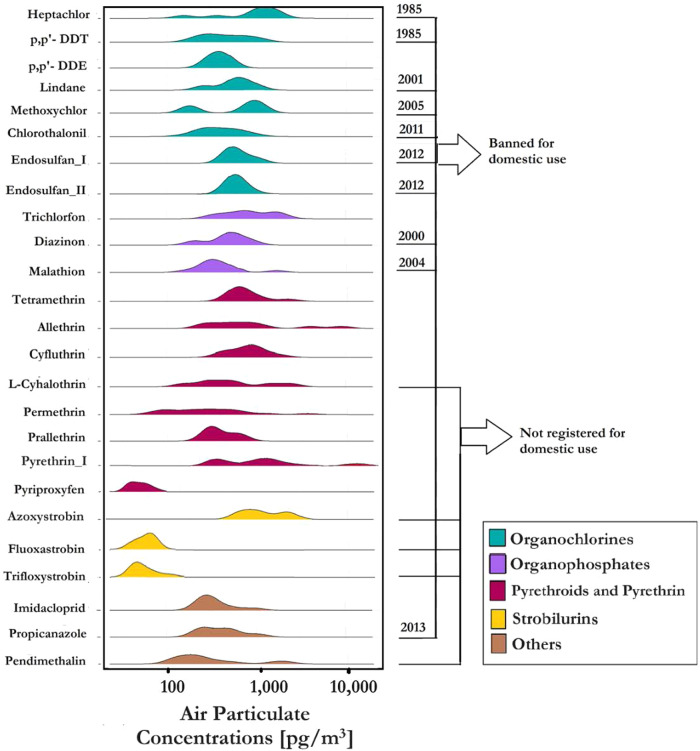
Range of particle-phase pesticide concentrations in social housing MURB units in Toronto, Canada, and the year in which a pesticide was restricted in Canada. Detected pesticides (above the method detection limit, Table S6) are shown on the *y*-axis, and ranges of the pesticide air concentrations in the particle phase that were above the detection limit are indicated on the *x*-axis. The details of the detection frequencies and maximum concentrations are presented in Table S6.

Canada, as a signatory to the Stockholm Convention, has complied with restrictions to the nine POPs OCPs [42, 54]. Regulatory re-evaluations in Canada have resulted in phasing out the use of almost all OPPs and carbamates for residential indoor [55]. The Pest Management Regulatory Agency (PMRA) of Canada also restricted some indoor uses of PYRs. For example, the use of tetramethrin for indoor perimeter and broadcast treatments has been canceled due to potential health impacts for humans, especially children [56]. A summary of these restrictions is presented in Fig. 1 [55, 57, 58].

Our goal was to measure the concentrations and thus exposures (e.g., inhalation) of low-SES households living in social housing located in Toronto, Canada to current-use and legacy pesticides in indoor air and to explore some factors related to those exposures. This paper aims to fill a data gap of residential exposures to current-use and legacy pesticide exposures amongst a vulnerable population, especially given the extremely limited data available for indoor pesticides in Canada [6].

## Materials and methods

Seven social housing MURBs, constructed in the 1970s at three sites in the City of Toronto, were monitored for pesticide concentrations. All buildings were located at a minimum of 65 km from any agricultural areas excluding home gardens. These buildings are typical of the social housing stock in Toronto. Our study was an extension of a larger study aimed at investigating particulate matter (PM) levels in social housing units before and after energy retrofits [59–61]. Thus, our sampling strategy was restricted to the collection of air PM only.

Retrofits involving water saving and energy saving (e.g., replacements of air handling units, boilers, and heating monitors) were designed, specifically for each unit, in order to reduce energy consumption and improve indoor air quality as well as enhance thermal comfort [62, 63]. The units were categorized according to their residents: seniors, families, and bachelors. Building characteristics and types are described in greater detail elsewhere [24].

### Sampling approach

Forty-six air filter samples taken from 46 units in winter 2017 in MURB social housing were analyzed. Wan et al. described the study design, sample collection, and storage procedures in detail [60]. In brief, each participant’s unit was fitted with an Amaircare XR-100 air cleaner equipped with 127 mm high-efficiency media (media used in HEPA filters) for a period of 1 week. Prior to and after deployments, all the portable air cleaners were cleaned using isopropyl wipes to avoid any cross-contamination. The portable air cleaners were placed on the wall, 30 cm from the ceiling, in the living room and/or according to the resident’s instructions in order to avoid inconveniencing residents and to minimize the potential for tampering (see Supplementary Information SI1, Fig. S1). During the 1-week sampling period, the median flow rate was 39.2 m^3^/day (see SI1 for details of the methods used to determine the flow rate). Preliminary door-to-door assessments of housing characteristics and resident behavior (like smoking) and visual checks took place in January and February 2015, before the samplers were deployed. After each visit from 2015 to 2017, an additional survey was administered. The full details are presented by Touchie et al. [64]. Briefly, the purpose of the survey was to assess the behavior of residents and potential changes in household characteristics and resident behavior such as tobacco smoking, operation of doors and windows, and use of rangehood exhaust or kitchen fan during cooking [59, 64]. Filters were analyzed after the retrofits for 28 target pesticides (considering, endosulfan I and II as well as α- and γ-chlordane as distinct compounds, and *p,p*′*-*DDE is the metabolite of *p,p*′*-*DDT not a pesticide), including both legacy and current use (Table S1).

### Analytical method

The extraction and cleanup process was described in detail by Wan et al. [60]. Each filter sample was divided in half, with one-half used for the analysis of 28 pesticides (Table S1). Filter samples and lab blanks consisting of glass fiber filters, which were added as one in every five samples for a total of nine, were spiked with six labeled pesticide surrogates (Table S2, Chromatographic Specialties Inc.) to monitor recoveries. Concentrations of target pesticides were also measured in five field blanks. Each filter sample was extracted by sonicating three times with 10 mL of hexane:acetone:dichloromethane (2:1:1, v:v:v), (HPLC grade, Fisher Scientific) for 20 min each time. Supernatants from each of the three extractions were combined and concentrated down to 1 mL under a steady stream of nitrogen in a Zymark Turbovap. Extracts were cleaned up using Florisil® SPE cartridges (Florisil® Superclean ENVI-Florisil SPE tubes, Supelco), then concentrated to 0.5 mL using a Zymark Turbovap and transferred to GC amber vials. Mirex (AccuStandard®) was then added as an internal standard (100 ng, Table S2). Analysis was done by gas chromatography–mass spectrometry (GC-MSD, Agilent 7890B GC and Agilent 5977A MSD) in both electron impact and chemical ionization modes. Instrumental parameters are given in SI4 and information on quantifier ions is presented in Tables S3, S4.

### Quality assurance/quality control

Labeled pesticide surrogates (Table S2) were spiked into samples and blanks prior to extraction to monitor recoveries throughout the analytical process. Recoveries of labeled compounds in samples ranged from 62 to 83%; results for all individual chemicals were recovery corrected. Data were blank corrected according to the criteria explained by Saini et al. [65] using the average lab and field blank values for each pesticide (values are presented in Table S5): no blank correction for an individual chemical when the blank concentration was <5% of the sample concentration; data were blank corrected when the blank concentration was 5–35%; and data were discarded if the blank concentration was >35% of values. Method detection limits (MDL, Table S6) were determined as the mean lab blank concentration (*n* = 9) plus three times the standard deviation. If a certain compound was not detected in blanks, then the Instrument limit of detection value was calculated using a signal-to-noise ratio of the compound in the lowest standard solution (~10:1). Concentrations in lab and field blanks were either <MDL or very low (Table S5); as such, the data did not require blank correction. Data < MDL were not substituted with ½ MDL for reporting and descriptive statistics since DF were all <50% [66].

#### Conversion of chemical mass to an integrated air concentration

Chemical mass on the air filters was converted to an integrated air-particle concentration using gravimetric analysis as well as the filter flow rate and the filtration efficiency according to Eq. 1:

1 $$C=\frac{{Mf}}{\eta {Qt}}$$

where *M* (g) is the total mass of PM captured by the filter, *f* (pg/g) is the contaminant concentration in the collected PM, and *η* is the filtration efficiency of the filter (assumed to be 100% due to the filter media and size of particles [67]), *Q* (m^3^/h) is the volumetric airflow rate through the portable air cleaner, and *t* (h) is the deployment time. Filter weights were recorded before and after deployment. Full details on airflow measurements and rates are provided by Wan et al. [60].

### Estimation of gas-phase concentrations

The sampling method used here measured particle-phase concentrations only. We estimated the equivalent gas-phase concentrations of pesticides using the Harner–Bidelman equation (Eq. 2), assuming chemical equilibrium between the phases [68]. Equation 2 was derived for outdoor particles, however, it has been used to estimate gas-particle partitioning in indoor environments as well [69, 70].

2 $$\log {Kp}=\log {Koa}\,+\log {fom}-11.91$$

where log *Kp* is the log transformation of the airborne particle-gas partition coefficient, log *Koa* is the log transformation of the octanol/air partition coefficient, *Koa* (unitless), ${fom}$ is the fraction of organic matter of the particles (unitless). The value for *fom* was consider to be 0.4 [71, 72]. Values of *Koa* were taken from OPERA 2.6 obtained through the CompTox Chemicals Dashboard (US EPA, 2023) (Fig. S2) because its estimates were found to have the least bias in comparison to other estimation methods [73]. We also obtained *Koa* experimental values and those estimated using Kowwin/HENRYWIN using EPISuite [74].

Gas-phase concentrations (*Cg*, pg/m^3^) were calculated using Eq. 3.

3 $${Cg}={Cp}/{Kp}$$

where *Cp* is the particle-phase concentration (pg/µg) and *Kp* has units of m^3^/µg.

### Data analysis

Since all the detected pesticides had DF of ≤50%, values < MDL were not imputed or replaced [66]. The Chi-square test was used to assess the normality of untransformed and log-transformed data. The Chi-square test indicated the non-normality of both untransformed and log-transformed data, which led us to use non-parametric statistical tests. Spearman rank correlation (*ρ*) for pesticides was used to evaluate co-usage of pesticides. The Mann–Whitney Wilcoxon test was used to test for significant differences between concentrations of tobacco-related pesticides in smoking vs non-smoking units, with significance established at *p* < 0.05. Correlations between pesticides were evaluated using Spearman’s rank correlation (*ρ*). Microsoft Excel 365 (Version 2202) and R (Version 4.1.2) were employed to obtain descriptive statistics.

## Results and discussion

### Detection frequencies

Detected pesticides from the 46 units sampled belonged to the OCPs, OPPs, PYRs, and strobilurins (STRs) classes and pendimethalin. In total, 24 out of 28 target pesticides were detected, with at least 1 pesticide detected in 89% of units. DF% ranged from 0 to 50% for OCPs, 11 to 24% for OPPs, 7 to 48% for PYRs, 7 to 22% for STRs, 22% for imidacloprid, 15% for propiconazole, and 41% for pendimethalin (see Table S6). Some variations in DF% of current-use pesticides can be explained through their availability in products containing pesticides as active ingredients. Of 2367 domestic products (referring to products purchased over-the-counter for personal use in and around residential settings) registered for use in Canada, pyrethrin I (DF = 48%) and permethrin (DF = 44%) were used in 367 and 340, respectively, while prallethrin (DF = 6.5%) is found in only 3 [75].

### Concentrations

Concentrations of pesticides in the particle phase (filters), as well as estimated gas phase and total concentrations, are presented in Fig. S3 and Tables S6, S8 for each pesticide group, based on *Koa* values from OPERA. The maximum values for gas-phase concentrations and sum of detected pesticides for each chemical group (i.e., Σ_8_OCPs, Σ_3_OPPs, Σ_8_PYRs, and Σ_3_STRs) obtained using experimental and estimated values of *Koa* from EPISuite are listed in Tables S7 and S8, respectively. We report particle-phase concentrations measured and compare the total air concentrations calculated here (using OPERA-based estimates) with air concentrations in the limited number of reports of pesticide air concentrations not related to agriculture and the few studies on low-SES families [26, 31, 76–78] (Table S9). It is important to note that this comparison is approximate due to the differences in sampling methods and years of study. To our knowledge, reported data here are the first Canadian results for pesticides in indoor air other than legacy organochlorines.

#### Organochlorine pesticides (OCPs)

In the particle phase, the maximum concentration of detected Σ_8_OCPs was 4400 pg/m^3^ (Table S8). The most abundant OCP was heptachlor (restricted in 1985) had a maximum concentration of 2600 pg/m^3^ followed by p,p′-DDT (restricted in 1985) with a maximum of 1400 pg/m^3^ [57]. Chlorothalonil, with a maximum concentration of 1200 pg/m^3^, is a pesticide used in paints as an anti-bacterial and fungal preservative. It had a DF of 50% despite the suspension of its registration for use in domestic applications in 2011 [55]. The relatively high DF’s and concentrations of legacy OCPs suggest the intensive use of OCPs in the past as well as their persistence indoors [6].

Previous studies have shown a positive correlation between building age and legacy OCP concentrations [6, 79]. Past uses of OCPs were for controlling pests indoors and specifically for lindane, as a treatment for lice which occurs with a higher frequency in lower than higher SES households [80, 81]. The maximum concentration of lindane was 990 pg/m^3^.

For total particle- plus gas-phase concentrations, heptachlor had the highest concentrations with a maximum of 443,000 pg/m^3^. Estimated maximum of total air concentrations of Σ_8_OCPs based on other range *Koa* values are presented in Table S8. Concentrations of heptachlor, lindane, chlorothalonil, and endosulfan I were 2 (chlorothalonil) to 11 (endosulfan I) times higher than those found in other studies in residential settings of both high to low-income households in the United States and France, where measurements were made up to 30 years ago [77, 82–84].

#### Organophosphate pesticides (OPPs)

The maximum of the sum of three the OPPs in the particle phase (Σ_3_OPPs), malathion, trichlorfon, and diazinon, was 3600 pg/m^3^. Among these, only malathion is currently registered in Canada for use in residential settings [55]. Trichlorfon had the highest particle-phase concentration in OPPs class with the maximum of 3600 pg/m^3^. In Canada, trichlorfon has been used as a technically active product to formulate other pest control products, such as those used to control non-resistant strains of flies and cockroaches [55]. Malathion, registered for residential use as a rodenticide, had a maximum concentration of 2800 pg/m^3^.

The maximum total (gas + particle) air concentration of Σ_3_OPPs was 77,000 pg/m^3^ (60,000–200,000 pg/m^3^ based on *Koa* values from EPISuite). DFs of 11–24% and air concentrations of OPPs were less than OCPs (DFs of 0–50%) which can likely be explained by the greater persistence of OCPs [85].

Concentrations of diazinon and malathion reported here were higher than those measured ~20 years ago in low-SES households in South Texas and Boston (only diazinon is reported) [26, 78]. We measured lower levels of diazinon than reported concentrations in studies of low to middle-SES families in New York and Northern California (we were unable to find more recent reports in the literature) [76, 77].

#### Pyrethroids/pyrethrins (PYRs)

PYRs are the most common pesticide for controlling bed bugs in many countries, but few studies have measured their levels in indoor air [86, 87]. These are the first data from Canada on indoor PYR concentrations.

In the particle phase, the maximum of $\,{\sum }_{8}{PYRs}$ was 36,000 pg/m^3^. Pyrethrin I was most frequently detected (DF% = 48) with highest maximum of all pesticides measured at 32,000 pg/m^3^. Pyrethrin I has been registered in Canada to control bed bugs, cockroaches, flying insects, and pests on pets [55, 88]. Also, pyrethrin I is considered a first-line treatment for head lice in Canada [89]. Considering that living in social housing carries a higher risk of bedbug infestation and head lice [80, 81], we anticipated high concentrations of pyrethrin I. To our knowledge, only one study reported air concentration of pyrethrin I in a residential setting, but not social housing. We observed higher concentrations compared to this literature value [90].

Allethrin also had relatively high concentrations, with the second-highest maximum particle-phase concentration of 16,000 pg/m^3^, followed by permethrin (maximum of 14,000 pg/m^3^). Allethrin and permethrin are commonly used in residential buildings. Similarly to pyrethrin I, permethrin is used in the treatment of head lice in Canada [89]. L-cyhalothrin was detected with a maximum concentration of 6000 pg/m^3^. While L-cyhalothrin has not been registered for domestic use in Canada, it has been approved for commercial use to preserve wood against carpenter ants [55, 91].

The maximum total air concentration of ${\sum }_{8}{PYRs}$ was 740,000 pg/m^3^ (110,000–270,000 based on EPISuite values of *Koa*). Concentrations of allethrin and permethrin here (maximum 406,000 pg/m^3^ and 14,500 pg/m^3^, respectively) were higher than those reported in studies of lower SES indoor air [26, 77, 78]. Whyatt et al., however, reported higher levels of permethrin in indoor air of low-SES homes in New York compared to our results (12 times higher) [76]. We measured a lower concentration range for tetramethrin with a maximum of 5300 pg/m^3^ compared with <MDL-63,000 in the study of Tulve et al. [92]. The Canadian PMRA canceled indoor broadcast and perimeter tetramethrin treatments due to health concerns [55, 56].

#### Strobilurins (STRs)

Although STR fungicides are not registered for domestic use in Canada, they may be used in some building materials such as mold-resistant wallboards [75, 93]. We measured relatively low particle-phase concentrations with a maximum ${\sum }_{3}{STRs}$ of 1200 pg/m^3^ and the total air concentration of ${\sum }_{3}{STRs}$ had maximum of 1300 pg/m^3^. Indoor air concentrations of STRs have not been previously reported.

#### Other pesticides

Imidacloprid, a neonicotinoid insecticide, is registered for use to control pests on pets in Canada [55]. Imidacloprid had a particle-phase maximum concentration of 930 pg/m^3^ and a maximum of 34,000 pg/m^3^ for total air.

Propiconazole fungicide is registered in Canada for use in building materials as a wood preservative [55]. We measured a maximum of 1100 pg/m^3^ in the particle phase and estimated maximum concentration of 2200 pg/m^3^ in total air.

Pendimethalin, a pesticide from the dinitroaniline class, had a maximum particle-phase concentration of 4400 pg/m^3^ and the maximum total air concentration was 9100 pg/m^3^. Pendimethalin is not registered for residential use in Canada although use related to tobacco may be a source as discussed below.

### Correlations among pesticides

Numerous pesticides were correlated with each other (Table S10). As expected, *p,p*′*-*DDT and *p,p*′-DDE were significantly correlated since *p,p*′-DDE is the metabolite of *p,p*′-DDT. Similarly, endosulfan I and endosulfan II were significantly correlated as they are two diastereoisomers that occurred together in technical grade endosulfan. The ratio of two diastereoisomers, endosulfan I: endosulfan II, varied from 2:1 to 7:3 depending on the technical mixture [94]. In our study, the ratio varied from 1:1 to 2:1.

Next, we looked for co-occurrence which could suggest the co-usage of pesticides as well as the use of multiple pesticides within a single pesticide product (see Upset plot, Fig. S4). For example, co-occurrence can occur because active ingredients can be co-formulated with other pesticides differing in their mechanisms of action, such as a mixture of pyriproxyfen with tetramethrin. Here we observed the correlations (*p* < 0.01) and co-occurrences of these pesticides (in 6 units) (Fig. S4 and Table S10) consistent with their combined formulation [75]. Significant correlations (*p* < 0.01) and co-occurrences were observed between OCPs such as *p,p*′-DDT with lindane (5 units) and heptachlor (6 units) suggesting their use over time or co-usage prior to their restriction. No co-occurrence was observed between OPPs except in 2 units where diazinon and malathion were detected.

We observed the high co-occurrences (8 units) between pyriproxyfen, imidacloprid, and permethrin which can be explained by the use of these three active pesticides in an insecticide product to control ticks, lice, and fleas on dogs [95]. Also, co-occurrences were observed between imidacloprid with L-cyhalothrin (4 units), prallethrin (4 units), and pyrethrin I (9 units). To our knowledge, there is no published Canadian information regarding the co-formulation of imidacloprid with L-cyhalothrin, prallethrin, and pyrethrin I. However, there are registered pesticides in other countries that contain mixtures of imidacloprid with L-cyhalothrin and prallethrin [96, 97]. Also we are not aware of a product that contains a mixture of pyrethrin I and imidacloprid. Application of these two insecticides could explain the observed co-occurrence since both are used to control bed bugs which are prevalent in social housing [86, 98]. We found a significant correlation (*p* < 0.01) and the most co-occurrence of permethrin and pyrethrin I (16 units) suggesting that they were used together, as were pyrethrin I and allethrin (7 units, *p* < 0.05), and permethrin and allethrin which showed a lower correlation (5 units, *p* < 0.05) [75]. Pendimethalin, permethrin, and chlorothalonil, which are used on tobacco crops, were also correlated and co-occurred in 9 units. Additional correlations and co-occurrences were observed between pesticides with no reported co-formulation such as permethrin with STRs (i.e., azoxystrobin, fluoxastrobin, and trifloxystrobin).

### Factors related to pesticide concentrations

#### Tobacco smoking

Tobacco cultivation and preparation are heavily reliant on pesticides. Levels of pesticides in tobacco decrease during harvesting, drying, and manufacturing of the final product. However, pesticide residues still remain in tobacco leaves [99]. Also, pesticides may be applied to tobacco leaves after harvesting [100]. Thus, tobacco leaves and smoke have both been found to contain pesticides [10].

In the Province of Ontario, more than half of the 12 largest social housing buildings do not follow smoke-free policies, putting residents at risk of second-hand smoke exposure [101]. Social housing MURBs that participated in our study were not smoke-free. Surveys were administered to residents in order to obtain information about their smoking habits. Also, in-unit checks were conducted during home visits in which evidence of smoking was sought [59, 64]. Residents smoked in 30% of units (14 out of 46) during the winter of 2017.

**Fig. 2:**
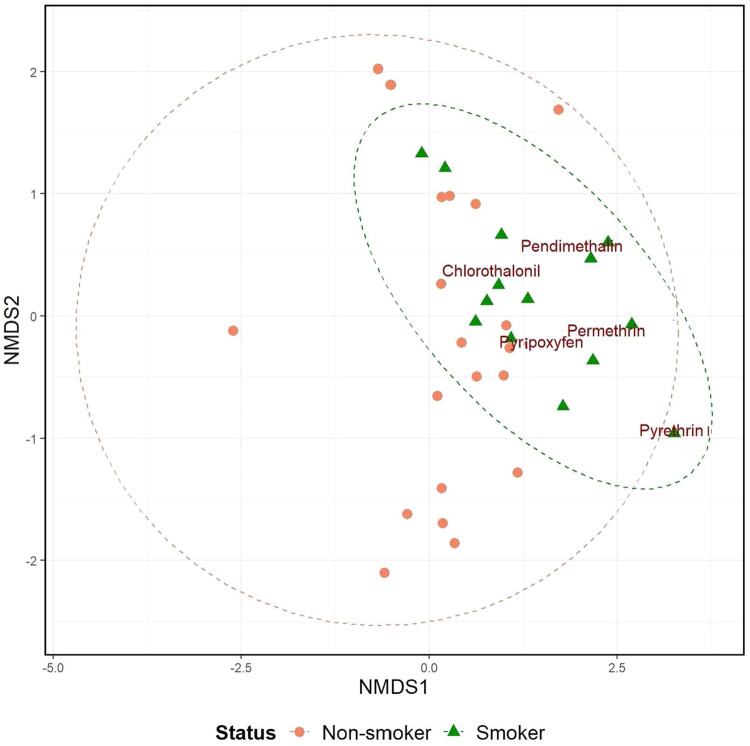
NMDS plot comparing pesticide concentrations with DF > 60% in units with reported tobacco smoking (green triangles) vs no evidence of tobacco smoking (orange dots). The stress level = 0.05 indicates a good fit.

We found an association between recorded smoking habits and exposure to specific pesticides, however, no relationships were found with other residents’ behaviors and household characteristics. The occurrence and concentrations of pesticides in smoking vs non-smoking households are shown in Fig. 2 using non-metric multidimensional scaling (NMDS). We found chlorothalonil, permethrin, pyriproxyfen, pyrethrin I, and pendimethalin had DF > 60% in units with reported smoking habits. The Mann–Whitney Wilcoxon test indicated significantly (*p* < 0.05) higher concentrations in smoking than non-smoking units for all the five pesticides (see Table S11 and Fig. S5, values < MDL for pesticides with DF > 60% were replaced by ½ MDL).

These five pesticides, except for pyriproxyfen, are active ingredients in herbicide and insecticide mixtures that can be applied to tobacco crops in Canada [55]. While pyriproxyfen is currently registered in Canada for pest control on pets, this insecticide has been referred to as a control agent for inhibiting the hatching of whitefly eggs on tobacco plants [55, 95]. These five herbicides were also detected in some units with non-smoking residents, which may have several possible explanations. We hypothesized that the presence of these herbicides/insecticides may be related to (1) other uses, such as the control of bed bugs or other insects; (2) migration of tobacco smoke from other units [102] as some residents in non-smoking units complained of tobacco odors [59]; and/or (3) changes in smoking habits (e.g., quitting, using electronic cigarettes) or in tenancy status (i.e., relocated of person who smoked). We note that no other significant relationships were found between information recorded in the survey and the pesticide data reported here.

#### Trends within and among buildings

**Fig. 3:**
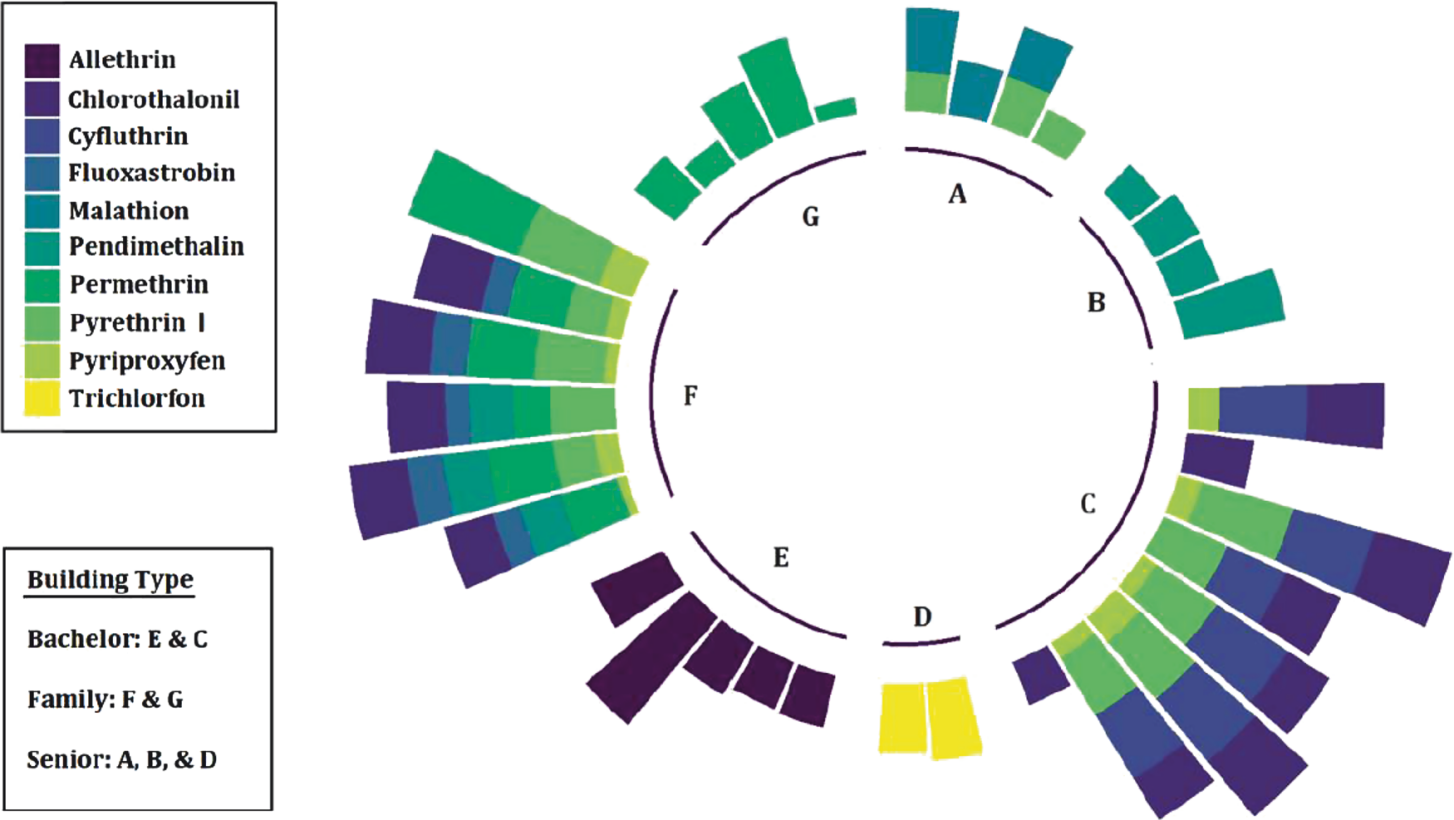
Particle-phase pesticide concentrations (log-transformed values for those compounds with DF > 50%) clustered according to buildings. Buildings are grouped as follows: Senior A, B, and D, Family = F and G, Bachelor = C and E. The size of each bar is proportional to concentration.

We examined the trends in pesticide concentrations (particle phase) within different buildings by considering pesticides with DF > 50% in units of a specific building. Figure 3 shows a clear pattern of pesticide concentrations among buildings (log_10_ transformed particle-phase concentrations with DF > 50%). The number of pesticides detected varied from five in building F to one in buildings B, D, E, and G. While trends were observed within a specific building, the number of, or specific pesticides detected, were not correlated with the unit type (i.e., senior, family, and bachelor). We hypothesized that the trends within buildings (e.g., buildings C and F) could result from the transfer of pesticides between units in these buildings, especially the more volatile pesticides with low *Koa* values, as occurs with other semi-volatile organic compounds [102–104]. In social housing MURBs, however, Wan et al. reported low inter-unit variations of PAHs suggesting low inter-unit transfer [60]. Thus, another explanation is that the similarity in pesticide profiles might be due to the pest eradication program implemented by building management throughout a specific building [105]. We were unable to verify this as we did not find records of pest abatement by building management or residents.

## Conclusions

Current-use pyrethroids had the highest DF and maximum concentrations in the particle phase of the 24 detected pesticides out of 28 total pesticides analyzed in air filters collected from 46 low-SES social housing units located in Toronto, Canada in 2017. Legacy OCPs and OPPs restricted for residential use as far back as 1985 (DDT) and up to 2012 (endosulfan) had DF of up to 50% and the highest estimated total air concentrations (e.g., heptachlor, lindane), reinforcing observations of high persistence indoors, on the order of decades. These are the first data reported for Canada for current use and recently restricted pesticides which prevented us from comparing to exposures to other SES groups. However, the levels reported here are higher relative to most of the literature values of indoor pesticide concentrations in low-SES residences. Significantly higher concentrations of five pesticides associated with tobacco production were measured in residences reporting smoking activity (chlorothalonil, permethrin, pyrethrin I, pyriproxyfen, and pendimethalin). Although STR fungicides are not registered for use indoors, their occurrence was likely related to the use of STR-treated building materials.

We were not aware of records regarding the application of pesticides by building managers or/and occupants in social housing MURBs. Consequently, occupants are unaware of and are unable to control their exposure to pesticides. These data add to the body of literature showing the exposure of residents to pesticides used indoors, even long after their application, that is more likely in social housing units which are prone to pest infestations and for which residents have limited ability to relocate.

This study had several limitations, notably the absence of data on pesticide applications by residents and/or building management. We were unable to compare the concentrations measured here for low-SES social housing households with values from higher SES households because of a lack of data from such household and a lack of resources to conduct an expanded study. Air sampling was conducted near the ceiling of each unit, far from the breathing zone to avoid inconveniencing residents and to minimize potential tampering. The strength of the study is the large number of pesticides analyzed from 46 households which presents a unique dataset for a vulnerable population and the only comprehensive data for indoor pesticides levels in Canada.

### Supplementary information

Supplementary Information
