# Supplementary material for: Widespread occurrence of pesticides in low-income housing
Source: J Expo Sci Environ Epidemiol. 2024 Jun 22;34(4):735–44. doi: 10.1038/s41370-024-00665-y (PMC11303252; doi:10.1038/s41370-024-00665-y)
Supplement: Supplementary file 1 — Supplementary Information [file 41370_2024_665_MOESM1_ESM.docx]

# ****Supporting Information****

**Widespread Occurrence of Pesticides in Low-income Canadian Homes**

Sara Vaezafshar^1^, Jeffrey A. Siegel^2,3^, Liisa Jantunen^1,4^, Miriam L. Diamond^1,3,5*^

^1^ Department of Earth Sciences, University of Toronto, Toronto, Ontario, Canada, M5S 3B1.

^2^ Department of Civil and Mineral Engineering, University of Toronto, Toronto, Ontario, Canada, M5S 1A4.

^3^ Dalla Lana School of Public Health, University of Toronto, Toronto, Ontario, Canada, M5T 3M7.

^4^Air Quality Processes Research, Environment and Climate Change Canada, Egbert, Canada, L0L 1N0.

^5^ School of Environment, University of Toronto, Toronto, Ontario, Canada, M5S 3E8.

^*^ Corresponding author: miriam.diamond@utoronto.ca

**SI1: Sampling Approach**

Portable air cleaners were deployed within 30 cm from the ceiling to be out-of-reach of residents (Figure S1). We note that this position was not within the breathing zone of adults or toddlers. Flow rates of each portable air cleaner were meticulously assessed both before and after deployment. This assessment was conducted within an airtight chamber, which was intricately connected to a calibrated fan (Duct Blaster 2 from The Energy Conservatory). The purpose of this connection was to nullify any pressure differentials within the chamber, thus ensuring precise measurements. Additionally, the chamber was outfitted with a micro-leakage meter (MLM, also from The Energy Conservatory), further enhancing the accuracy of our measurements. During the sampling, apartments were set up with a long-term monitoring package, consisting of a CO_2_ sensor (Onset HOBO U12-012) and a temperature probe (Onset TXCx-HD). After deployment, the filter media were individually stored at -20^o^C in anti-static bags until analysis (1). A plot study conducted by Wan et al. (1) confirmed low cross-contamination during the conditioning and sampling process.


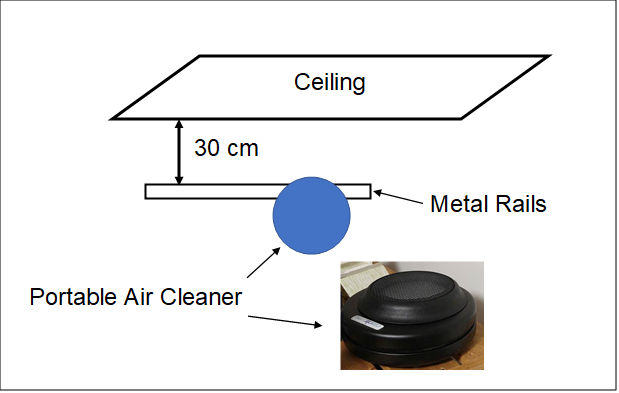


Figure S1. Portable air cleaner set-up in multi-residential buildings (MURBs) social housing. The portable air cleaner is highlighted in blue. The figure is adapted from Wan et al., (1).

**SI2: Compounds of Interest**

| Table S1. A summary of names, CAS numbers (CAS No.), molecular formulas (MF) and molecular weights (MW) of the target compounds.   \| **Compound Name** \| **CAS No.** \| **MF** \| **MW (g/mol)** \| \| --- \| --- \| --- \| --- \| \| Heptachlor \| 76-44-8 \| C_10_H_5_Cl_7_ \| 373.32 \| \| *p,p’*-DDT \| 50-29-3 \| C_14_H_9_Cl_5_ \| 354.49 \| \| *p,p’*-DDE \| 72-55-9 \| C_14_H_8_Cl­_4_ \| 318.03 \| \| Chlordane [Isomer mixture: α-Chlordane and γ-Chlordane] \| 57-74-9 \| C_10_H_6_Cl_8_ \| 409.78 \| \| Chlorpyrifos \| 2921-88-2 \| C_9_H_11_Cl_3_NO_3_PS \| 350.59 \| \| Lindane \| 58-89-9 \| C_6_H_6_Cl_6_ \| 290.83 \| \| Methoxychlor \| 72-43-5 \| C_16_H_15_Cl_3_O_2_ \| 345.65 \| \| Chlorthal-dimethyl \| 1861-32-1 \| C_10_H_6­_Cl_4_O_4_ \| 331.96 \| \| Chlorothalonil \| 1897-45-6 \| C_8_Cl_4_N_2_ \| 265.91 \| \| Endosulfan-I \| 959-98-8 \| C_9_H_6_Cl_6_O_3_S \| 406.93 \| \| Endosulfan-II \| 33213-65-9 \| C_9_H_6_Cl_6_O_3_S \| 406.93 \| \| Trichlorfon \| 52-68-6 \| C_4_H_8_Cl_3_O_4_P \| 257.44 \| \| Diazinon \| 333-41-5 \| C_12_H_21_N_2_O_3_PS \| 304.35 \| \| Malathion \| 121-75-5 \| C_10_H_19_O_6_PS_2_ \| 330.36 \| \| Tetramethrin \| 7696-12-0 \| C_19_H_25_NO_4_ \| 331.41 \| \| Allethrin \| 587-79-2 \| C_19_H_26_O_3_ \| 302.41 \| \| Cyfluthrin \| 68359-37-5 \| C_22_H_18_Cl_12_FNO_3_ \| 434.30 \| \| L-Cyhalothrin \| 91465-08-6 \| C_23_H_19_ClF_3_NO_3_ \| 449.90 \| \| Permethrin \| 52645-53-1 \| C_21_H_20_Cl_2_O_3_ \| 3921.28 \| \| Prallethrin \| 23031-36-9 \| C_19_H_24_O_3_ \| 300.4 \| \| Pyrethrin-I \| 121-21-1 \| C_21_H_28­_O_3_ \| 328.4 \| \| Pyriproxyfen \| 96737-68-1 \| C_20_H_19_NO_3_ \| 321.37 \| \| Azoxystrobin \| 131860-33-8 \| C_22_H_17_N_3_O_5_ \| 403.39 \| \| Fluoxastrobin \| 361377-29-9 \| C_21_H_16_ClFN_4_O_5_ \| 458.83 \| \| Trifloxystrobin \| 141517-21-7 \| C_20_H_19_F_3_N_2_O_4_ \| 408.37 \| \| Imidacloprid \| 138261-41-3 \| C_9_H_10_ClN_5_O_2_ \| 255.661 \| \| Propiconazole \| 60207-90-1 \| C_15_H_17_Cl_2_N_3_O_2_ \| 342.22 \| \| Pendimethalin \| 40487-42-1 \| C_13_H_19_N_3_O_4_ \| 281.308 \| |
| --- | --- | --- | --- | --- | --- | --- | --- | --- | --- | --- | --- | --- | --- | --- | --- | --- | --- | --- | --- | --- | --- | --- | --- | --- | --- | --- | --- | --- | --- | --- | --- | --- | --- | --- | --- | --- | --- | --- | --- | --- | --- | --- | --- | --- | --- | --- | --- | --- | --- | --- | --- | --- | --- | --- | --- | --- | --- | --- | --- | --- | --- | --- | --- | --- | --- | --- | --- | --- | --- | --- | --- | --- | --- | --- | --- | --- | --- | --- | --- | --- | --- | --- | --- | --- | --- | --- | --- | --- | --- | --- | --- | --- | --- | --- | --- | --- | --- | --- | --- | --- | --- | --- | --- | --- | --- | --- | --- | --- | --- | --- | --- | --- | --- | --- | --- | --- |

**SI3: Analytical Methods**

Table S2. Surrogate and internal standards used.

| **Compound Name** | **Type** | | | **Supplier** | **Mass added to each sample (ng)** |
| --- | --- | --- | --- | --- | --- |
| d_10_-chlorpyrifos | | Surrogate Standard | | Cambridge Isotope Laboratories (CIL), Inc | 90 |
| d_14_-trifluralin | | Surrogate Standard | | CIL, Inc | 100 |
| ^13^C-hexachlorobenzene | | Surrogate Standard | | CIL, Inc | 100 |
| d_5_-atrazine | | Surrogate Standard | | CIL, Inc | 100 |
| ^13^C-endosulfan-II | | Surrogate Standard | | CIL, Inc | 100 |
| d_6_- γ -HCH | | Surrogate Standard | | CIL, Inc | 100 |
| Mirex | | Internal Standard | | CIL, Inc | 100 |
|  | | |  | | |

**SI4: Instrumental Parameters**

**Pesticides Analyzed by GC-NCI-MS**

Pesticides presented in Table S3 were analyzed using an Agilent GC 7890-MSD 5977 operating in electron capture negative ionization (ECNI) mode in selective ion monitoring (SIM). Separation of target compounds were performed on a DB-5 MS column (Agilent Technologies, 30 m x 0.25 mm i.d. x 0.25 µm film thickness). The initial oven temperature program was 100°C with a hold for 2 min, then 5 °C/min to 220 °C hold for 5 min, 5 °C/min to 225 holds for 5 min, 15 °C/min to 300 for 5 min. The post-run was at 290 °C for 3 min. The injection volume was 1 µL at splitless mode with an inlet temperature of 275 °C. Both source and quad temperatures were 150 °C and the transfer line temperature was 290 °C. The helium carrier gas flow rate was 1.2 mL/min and methane was used as reagent gas.

**Pesticides Analyzed by GC-EI-MS**

All the pesticides listed in Table S4 except fluoxastrobin, trifloxystrobin, and azoxystrobin were analyzed using Agilent GC 6890 - MS 5975 systems operating in electron impact (EI) mode in SIM. The targeted compounds were separated on a 15 m DB-5 MS column (Agilent Technologies, 0.25 mm x 0.25 µm). The oven temperature program was initially 100°C hold for 2 min, then 5°C/min to 220°C hold for 5 min, 15 °C/min to 280 °C hold for 5 min, and 15 °C/min to 300 °C hold for 5 min. The post-run was 290°C for 3 min. The injection volume was 1 µL at splitless mode with an inlet temperature of 275 °C. The helium carrier gas flow rate was 1.2 mL/min. Transfer line, source, and quad temperatures were 320, 230, and 150 °C respectively. Fungicides including fluoxastrobin, trifloxystrobin, and azoxystrobin were analyzed using the same parameters as above except for the temperature program: initial at 80°C hold for 0.5 min, then 10°C/min to 100°C hold for 1 min, 25 °C/min to 280 °C hold for 1 min, 10 °C/min to 300 °C for 10 min. The post-run was at 290°C for 2 min.

**SI5: Quantifier and Qualifier Ions**

Table S3. List of the quantifier and qualifier ions in GC-ECNI-MS.

| **Compound Name** | **Quantifier Ion** | **Qualifier Ion** |
| --- | --- | --- |
| Heptachlor | 300 | 302 |
| *p,p’*-DDT | 248 | 250 |
| *p,p'*-DDE | 318 | 316 |
| α-Chlordane (cis) | 410 | 408/412 |
| γ-Chlordane (trans) | 410 | 408/412 |
| Chlorpyrifos | 313 | 315 |
| Lindane | 255 | 257 |
| Chlorothalonil | 266 | 264 |
| Chlorthal-dimethyl | 332 | 330 |
| Endosulfan-I | 406 | 404 |
| Endosulfan-II | 406 | 404 |
| Malathion | 157 | 172 |
| Pendimethalin | 281 | 282 |
| Mirex | 404 | / |
| ^13^C-hexachlorobenzene | 290 | 336 |
| ^13^C-endosulfan-II | 413 | 404 |
| d_10_-chrorpyrifos | 323 | 315 |
| d_14_-trifluralin | 349 | / |
| d_6_-γ-HCH | 261 | 263 |

Table S4. List of the quantifier and qualifier ions in GC-NEI-MS.

| **Compound Name** | **Quantifier Ion** | **Qualifier Ion** |
| --- | --- | --- |
| Methoxychlor | 227 | 228/212 |
| Trichlorfon | 109 | 145/112 |
| Diazinon | 179 | 137 |
| Tetramethrin | 181 | 265/125 |
| Allethrin | 167 | 134/168 |
| Cyfluthrin | 163 | 206/226/165 |
| L-Cyhalothrin | 181 | 197/208 |
| Permethrin | 182 | 163/91/165 |
| Prallethrin | 123 | 81/77 |
| Pyrethrin-I | 107 | 123 |
| Pyriproxyfen | 136 | / |
| Azoxystrobin | 344 | / |
| Fluoxastrobin | 188 | 219 |
| Trifloxystrobin | 222 | 131 |
| Imidacloprid | 168 | 211/126/99 |
| Propiconazole | 173 | 259/261 |
| Mirex | 272 | 274 |
| d_5_-atrazine | 205 | / |

**SI6: Koa values**

**
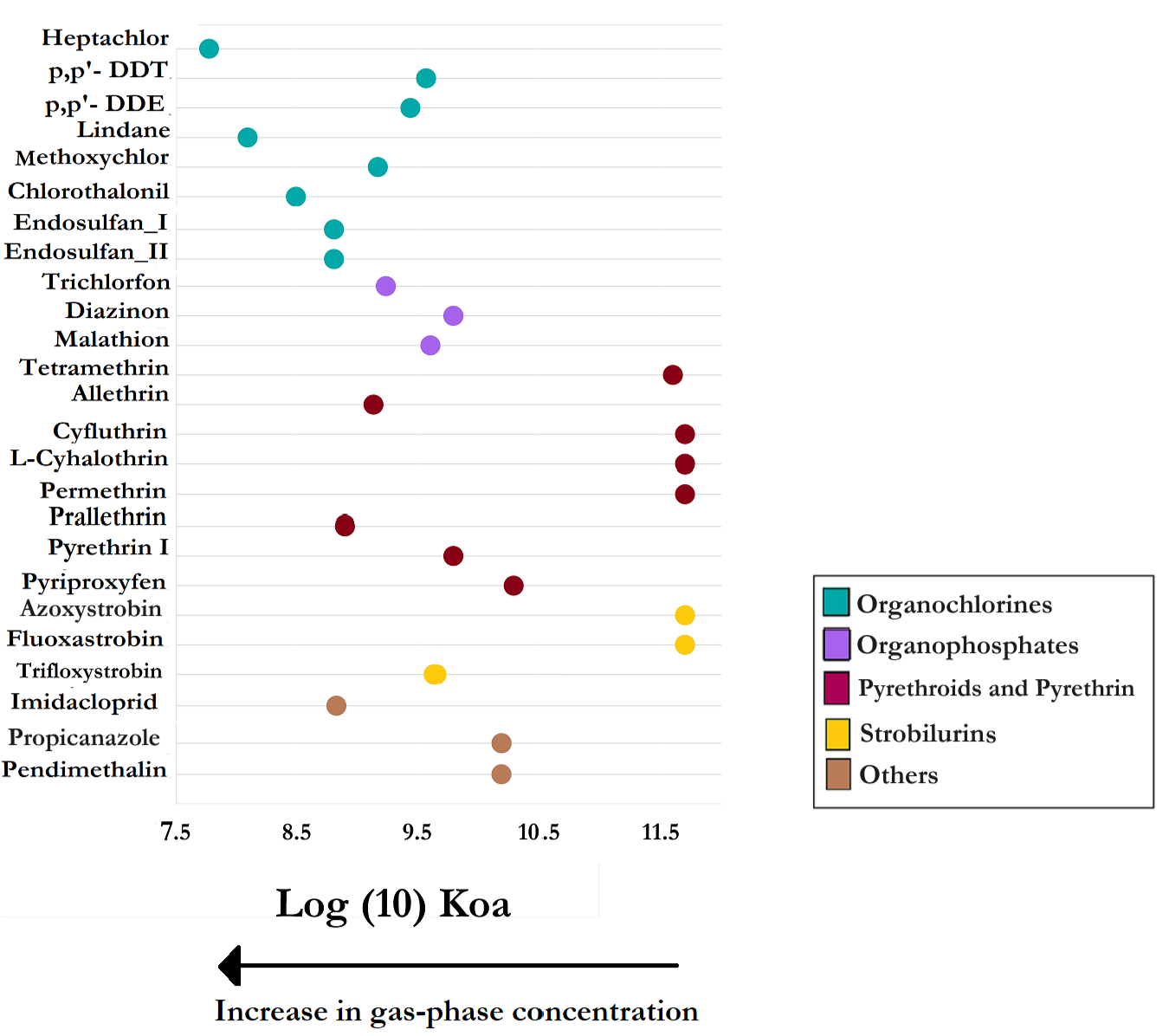
**

Figure S2. Koa values for pesticides measured, taken from Opera 2.6 accessed through the [CompTox Chemicals Dashboard](https://comptox.epa.gov/dashboard/) (EPA, n.d.)

**SI7: Laboratory and Field Blanks**

Table S5. Concentrations (ng) of target pesticides in 9 laboratory blanks (LB) and field blanks (FB) were used to calculate the Method Detection Limit (MDL) for each compound (mean blank concentrations + 3 x lab blank standard deviation). If a compound was not detected (N.D.) in blanks, then the Instrument Limit of detection (IDL) value was calculated. IDL was the signal-to-noise ratio of the pesticide in the lowest standard solution (usually about 1:10).

| **Compounds** | **LB 1** | **LB 2** | **LB 3** | **LB 4** | **LB 5** | **LB 6** | **LB 7** | **LB 8** | **LB 9** | **FB 1** | **FB 2** | **FB 3** | **FB 4** | **FB 5** |
| --- | --- | --- | --- | --- | --- | --- | --- | --- | --- | --- | --- | --- | --- | --- |
| Heptachlor | N.D. | N.D. | N.D. | N.D. | N.D. | N.D. | N.D. | N.D. | N.D. | N.D. | N.D. | N.D. | N.D. | N.D. |
| *p,p’*-DDT | 10 | 13 | 13 | 11 | 10 | 16 | 12 | 15 | 11 | 13 | 13 | 12 | 13 | 14 |
| *p,p’*- DDE | 4.4 | 3.8 | 3.4 | N.D. | 4.0 | N.D. | 4.6 | 4.4 | N.D. | 2.4 | 2.5 | 3.1 | 2.4 | 3.5 |
| α-Chlordane | N.D. | N.D. | N.D. | N.D. | N.D. | N.D. | N.D. | N.D. | N.D. | N.D. | N.D. | N.D. | N.D. | N.D. |
| γ-Chlordane | N.D. | N.D. | N.D. | N.D. | N.D. | N.D. | N.D. | N.D. | N.D. | N.D. | N.D. | N.D. | N.D. | N.D. |
| Chlorpyrifos | 2.9 | 4.2 | 4.8 | N.D. | 3.8 | N.D. | 3.8 | 3.8 | 4.8 | 2.1 | 3.0 | 2.4 | 2.4 | 3.2 |
| Lindane | N.D. | N.D. | N.D. | N.D. | N.D. | N.D. | N.D. | N.D. | N.D. | N.D. | N.D. | N.D. | N.D. | N.D. |
| Methoxychlor | 15 | 18 | 18 | 15 | 13 | 22 | 14 | 11 | 15 | 14 | 14 | 12 | 11 | 15 |
| Chlorthal-dimethyl | N.D. | N.D. | N.D. | N.D. | N.D. | N.D. | N.D. | N.D. | N.D. | N.D. | N.D. | N.D. | N.D. | N.D. |
| Chlorothalonil | N.D. | N.D. | N.D. | N.D. | N.D. | N.D. | N.D. | N.D. | N.D. | N.D. | N.D. | N.D. | N.D. | N.D. |
| Endosulfan-I | N.D. | 5.0 | 5.3 | N.D. | 5.0 | N.D. | 5.5 | N.D. | N.D. | N.D. | N.D. | N.D. | N.D. | N.D. |
| Endosulfan-II | N.D. | 4.8 | 4.0 | N.D. | 4.8 | N.D. | 5.0 | N.D. | N.D. | N.D. | N.D. | N.D. | N.D. | N.D. |
| Trichlorfon | 22 | 27 | 26 | 27 | 23 | 24 | 26 | 26 | 27 | 24 | 23 | 21 | 26 | 24 |
| Diazinon | 18 | 16 | 21 | 18 | 6.4 | 24 | 18 | 15 | 20 | 19 | 15 | 18 | 11 | 18 |
| Malathion | N.D. | N.D. | N.D. | N.D. | N.D. | N.D. | N.D. | N.D. | N.D. | N.D. | N.D. | N.D. | N.D. | N.D. |
| Tetramethrin | 37 | 59 | 53 | N.D. | 26 | N.D. | 51 | N.D. | 34 | 33 | 32 | 35 | 34 | 34 |
| Allethrin | 25 | 21 | 15 | 25 | 11 | 33 | 26 | 17 | 29 | 62 | 50 | 72 | 41 | 57 |
| Cyfluthrin | 109 | 99 | 104 | 100 | 101 | 98 | 116 | 103 | 95 | 96 | 109 | 83 | 99 | 85 |
| L-cyhalothrin | 13 | 16 | 17 | 14 | 13 | 20 | 13 | 16 | 18 | 15 | 17 | 15 | 14 | 14 |

| Table S5. Continued | |  |  |  |  |  |  |  |  |  |  |  |  |  |
| --- | --- | --- | --- | --- | --- | --- | --- | --- | --- | --- | --- | --- | --- | --- |
|  |  |  |  |  |  |  |  |  |  |  |  |  |  |  |
| Compound | **LB 1** | **LB 2** | **LB 3** | **LB 4** | **LB 5** | **LB 6** | **LB 7** | **LB 8** | **LB 9** | **FB 1** | **FB 2** | **FB 3** | **FB 4** | **FB 5** |
| Permethrin | 1.2 | 2.3 | 1.5 | 1.4 | 2.0 | 1.2 | 1.5 | 1.2 | 1.6 | 4.2 | 3.8 | 4.0 | 4.1 | 3.5 |
| Prallethrin | 1.2 | 1.1 | 0.8 | 0.7 | 0.5 | 0.4 | 0.7 | 0.8 | 0.3 | 2.6 | 2.3 | 3.0 | 1.8 | 2.0 |
| Pyrethrin I | 0.4 | 0.9 | 0.4 | 0.3 | 0.7 | 0.6 | 0.2 | 0.2 | 0.1 | 1.7 | 1.5 | 1.7 | 2.3 | 1.2 |
| Pyriproxyfen | N.D. | N.D. | N.D. | N.D. | N.D. | N.D. | N.D. | N.D. | N.D. | N.D. | N.D. | N.D. | N.D. | N.D. |
| Azoxystrobin | 6.6 | 5.4 | 6.9 | 5.3 | 4.5 | 8.3 | 7.4 | 5.7 | 3.5 | 0.9 | 0.6 | 1.2 | 0.7 | 1.0 |
| Fluoxastrobin | N.D. | N.D. | N.D. | 4.1 | 3.3 | 4.2 | N.D. | 4.2 | N.D. | N.D. | N.D. | N.D. | N.D. | N.D. |
| Trifloxystrobin | 4.8 | 4.5 | 6.0 | 4.9 | 2.5 | 8.0 | 5.4 | 7.1 | 5.0 | 8.0 | 5.5 | 3.2 | 5.7 | 8.0 |
| Imidacloprid | 25 | 23 | 20 | 26 | 13 | 39 | 27 | 13 | 27 | 42 | 38 | 31 | 40 | 32 |
| Propiconazole | 30 | 25 | 16 | 26 | 10 | 38 | 31 | 27 | 32 | 34 | 32 | 38 | 30 | 32 |
| Pendimethalin | N.D. | N.D. | N.D. | N.D. | N.D. | N.D. | N.D. | N.D. | N.D. | N.D. | N.D. | N.D. | N.D. | N.D. |

**SI8: Descriptive Statistics**

Table S6. Detection frequencies (D.F., %) and maximum concentrations (Max), and method detection limits (MDL) for target pesticides in air particle samples collected from social housing MURB units (n=46). Gas-phase concentrations were estimated using the relationship of Harner and Bidleman (2) with values of Koa from OPERA 2.6 from the CompTox Chemicals Dashboard (3). Minimum values for all the pesticides were below MDL. All the detected pesticides had DF≤50%. Thus, values < MDL were not imputed with ½ MDL and median values are not reported (4). The Method detection limit (MDL) for each compound was calculated as described above. IDL was used for compounds marked with “*”.

|  |  |  |  |  | **Particle phase (pg/m^3^)** | **Estimated Gas phase (pg/m^3^)** | **Total air concentration (pg/m^3^)** |
| --- | --- | --- | --- | --- | --- | --- | --- |
|  | **Compounds** | **D.F. (%)** | **MDL (ng)** |  | **Max** | **Max** | **Max** |
| Heptachlor | | 17 | 1.0*  18  5.7  3.2*  3.2*  6.0  2.7*  23  6.7*  2.0*  9.8  5.8  31  29  1.4*  68  85 | | 2,600 | 440,000 | 443,000 |
| *p,p’*-DDT | | 29 |  |  | 1,400 | 4,600 | 6,000 |
| *p,p’*- DDE | | 7 |  |  | 240 | 1,200 | 1,300 |
| α-Chlordane | | 0 |  |  | - | - | - |
| γ-Chlordane | | 0 |  |  | - | - | - |
| Chlorpyrifos | | 0 |  |  | - | - | - |
| Lindane | | 11 |  |  | 990 | 300,000 | 300,000 |
| Methoxychlor | | 7 |  |  | 1,200 | 13,000 | 14,000 |
| Chlorthal-dimethyl | | 0 |  |  | - | - | - |
| Chlorothalonil | | 50 |  |  | 1,200 | 64,000 | 65,000 |
| Endosulfan-I | | 17 |  |  | 1,100 | 150,000 | 151,000 |
| Endosulfan-II | | 17 |  |  | 760 | 160,000 | 161,000 |
| Trichlorfon | | 13 |  |  | 3,600 | 77,000 | 81,000 |
| Diazinon | | 11 |  |  | 760 | 19,000 | 20,000 |
| Malathion | | 24 |  |  | 2,800 | 57,000 | 60,000 |
| Tetramethrin | | 22 |  |  | 5,000 | 270 | 5,300 |
| Allethrin | | 22 |  |  | 16,000 | 390,000 | 406,000 |

Table S6. Continued

|  |  |  | **Particle phase (pg/m^3^)** | **Estimated Gas phase (pg/m^3^)** | **Total air concentration (pg/m^3^)** |
| --- | --- | --- | --- | --- | --- |
| **Compounds** | **D.F. (%)** | **MDL (ng)** | **Max** | **Max** | **Max** |
| Cyfluthrin | 20 | 124 | 3,300  6000  14,000  380  32,000  47  1,100  94  120  930  1,100  4,400 | 310 | 3,600 |
| L-cyhalothrin | 15 | 21 |  | 250 | 6,250 |
| Permethrin | 44 | 5.8 |  | 450 | 14,500 |
| Prallethrin | 7 | 3.6 |  | 31,000 | 31,500 |
| Pyrethrin I | 48 | 2.6 |  | 310,000 | 330,000 |
| Pyriproxyfen | 41 | 2.0* |  | 180 | 230 |
| Azoxystrobin | 9 | 11 |  | 63 | 1,200 |
| Fluoxastrobin | 22 | 5.1 |  | 2.2 | 96 |
| Trifloxystrobin | 7 | 10 |  | 310 | 430 |
| Imidacloprid | 22 | 54 |  | 33,000 | 34,000 |
| Propiconazole | 15 | 49 |  | 1,100 | 2,200 |
| Pendimethalin | 41 | 3.1* |  | 4,700 | 9,100 |

| Table S7. Particle phase concentration (pg/g) [maximum] of detected pesticides in collected samples from social housing MURB units (n=46), calculated gas- phase concentrations (pg/m^3^) [maximum] using three values for log Koa from OPERA 2.6 (Comptox Chemicals Dashboard), experimental values from EPISuite and estimated values from Kowwin/HENRYWIN-EPISuite (5). The default reported values for gas-phase concentrations in the current study were estimated using OPERA. | | | | | | | | | | | | | | | |  |
| --- | --- | --- | --- | --- | --- | --- | --- | --- | --- | --- | --- | --- | --- | --- | --- | --- |
| **Compound** | **Particle phase**  **Concentration (ug/g)** | **log Koa ^1^** | **Log Kp ^2^** | **Cg (pg/m^3^)** | **log Koa^3^** | **Log Kp** | | | **Cg (pg/m^3^)** | **Log Koa^4^** | | **Log Kp** | | **Cg (pg/m^3^)** | |  |
| Heptachlor | 13 | 7.77 | -4.54 | 440,000 | 7.39 | | -4.92 | 1,100,000 | | 8.00 | -4.30 | | 260,000 | | |  |
| p,p’-DDT | 8.3 | 9.57 | -2.74 | 4,600 | 10.2 | | -1.93 | 710 | | 9.99 | -2.31 | | 1,700 | | |  |
| p,p’- DDE | 1.7 | 9.44 | -2.87 | 1,200 | 9.28 | | -3.03 | 1,700 | | 8.84 | -3.47 | | 4,600 | | |  |
| Lindane | 18 | 8.09 | -4.22 | 300,000 | 7.82 | | -4.49 | 560,000 | | 6.24 | -6.07 | | 21,000,000 | | |  |
| Methoxychlor | 9.6 | 9.17 | -3.14 | 13,000 | 10.6 | | -1.70 | 480 | | 11.1 | -1.24 | | 170 | | |  |
| Chlorothalonil | 9.7 | 8.49 | -3.84 | 64,000 | 7.14 | | -5.17 | 1,400,000 | | 8.87 | -3.44 | | 27,000 | | |  |
| Endosulfan-I | 49 | 8.81 | -3.50 | 150,000 | 6.41 | | -5.90 | 38,000,000 | | 8.93 | -3.38 | | 120,000 | | |  |
| Endosulfan-II | 52 | 8.81 | -3.50 | 160,000 | 6.41 | | -5.90 | 41,000,000 | | 8.93 | -3.38 | | 120,000 | | |  |
| Trichlorfon | 66 | 9.24 | -3.07 | 77,000 | 9.86 | | -2.45 | 18,000 | | 12.0 | -0.33 | | 140 | | |  |
| Diazinon | 59 | 9.83 | -2.51 | 19,000 | 9.15 | | -3.16 | 85,000 | | 9.31 | -3.00 | | 60,000 | | |  |
| Malathion | 120 | 9.61 | -2.70 | 57,000 | 9.06 | | -3.25 | 200,000 | | 9.76 | -2.55 | | 41,000 | | |  |
| Tetramethrin | 52 | 11.6 | -0.71 | 270 | 8.89 | | -3.42 | 140,000 | | 12.2 | -0.12 | | 69 | | |  |
| Allethrin | 280 | 9.16 | -2.16 | 390,000 | 10.2 | | -2.16 | 40,000 | | 10.1 | -2.19 | | 43,000 | | |  |
| Cyfluthrin | 78 | 11.7 | -0.61 | 310 | 11.8 | | -0.51 | 250 | | 10.16 | -2.14 | | 11,000 | | |  |
| L-cyhalothrin | 62 | 11.7 | -0.61 | 250 | 11.2 | | -1.09 | 750 | | 10.11 | -2.20 | | 9,700 | | |  |
| Permethrin | 110 | 11.7 | -0.61 | 450 | 10.6 | | -1.69 | 5,500 | | 12.4 | 0.05 | | 99 | | |  |
| Prallethrin | 12 | 8.90 | -3.41 | 31,000 | 9.87 | | -2.44 | 3,400 | | 10.7 | -2.05 | | 1,400 | | |  |
| Pyrethrin I | 950 | 9.83 | -2.51 | 310,000 | 10.4 | | -1.91 | 77,000 | | 10.8 | -1.53 | | 32,000 | | |  |
| Pyriproxyfen | 1.7 | 10.3 | -2.01 | 180 | 13.1 | | 0.831 | 0.250 | | 13.1 | 0.82 | | 0.260 | | |  |
| Azoxystrobin | 15 | 11.7 | -0.61 | 6.3 | 14.0 | | 1.72 | 0.29 | | 13.1 | 1.72 | | 2.71 | | |  |
| Fluoxastrobin | 0.55 | 11.7 | -0.61 | 2.2 | No Value | | - | - | | - | - | | - | | |  |
| Trifloxystrobin | 0.58 | 9.58 | -1.81 | 300 | 9.86 | | -2.45 | 160 | | 12.0 | -0.33 | | 1.2 | | |  |
| Imidacloprid | 11 | 8.83 | -3.48 | 33,000 | 13.7 | | 1.43 | 0.40 | | 11.9 | -0.38 | | 26 | | |  |
| Propiconazole | 8.7 | 10.2 | -2.11 | 1,100 | 10.9 | | -1.44 | 240 | | 11.4 | -0.92 | | 73 | | |  |
| Pendimethalin | 37 | 10.2 | -2.11 | 4700 | 9.64 | | -2.67 | 17,000 | | 9.05 | -3.26 | | 67,000 | | |  |
| ^1^ Comptox Chemicals Dashboard (2), ^2^ log Kp= log Koa + log fom-11.91, ^3^Estimated using experimental value-EPI suite (5), ^4^Estimated from Kowwin/HENRYWIN-EPI suite | | | | | | | | | | | | | | |  | |

|  | **Particle phase (pg/m^3^)** | **^1^ Total air (pg/m^3^)** | **^2^ Total air (pg/m^3^)** | **^3^ Total air (pg/m^3^)** |
| --- | --- | --- | --- | --- |
| $\sum_{8} OCPs$ | 4,400 | 440,000 | 79,000,000 | 2,200,000 |
|  |  |  |  |  |
| $\sum_{3} OPPs$ | 3,600 | 77,000 | 200,000 | 60,000 |
|  |  |  |  |  |
| $\sum_{8} PYRs$ | 36,000 | 740,000 | 270,000 | 110,000 |
|  |  |  |  |  |
| $\sum_{3} STRs$ | 1,200 | 1,300 | 1,200 | 1,200 |
| Total air (pg/m3) were calculated using three values for *Koa*: ^1^ Comptox chemical dashboard (2), ^2^Estimated using experimental value-EPI suite (5), ^3^Estimated from Kowwin/HENRYWIN-EPI suite. | | | | |

Table S8. The maximum (Max) particle phase and total air (gas + particle phase) for summation of target pesticides in each chemical group, OCPs (organochlorines), OPPs (organophosphates), PYRs (pyrethroids/pyrethrins) and STRs (strobilurins).

**SI9: Total air concentrations**


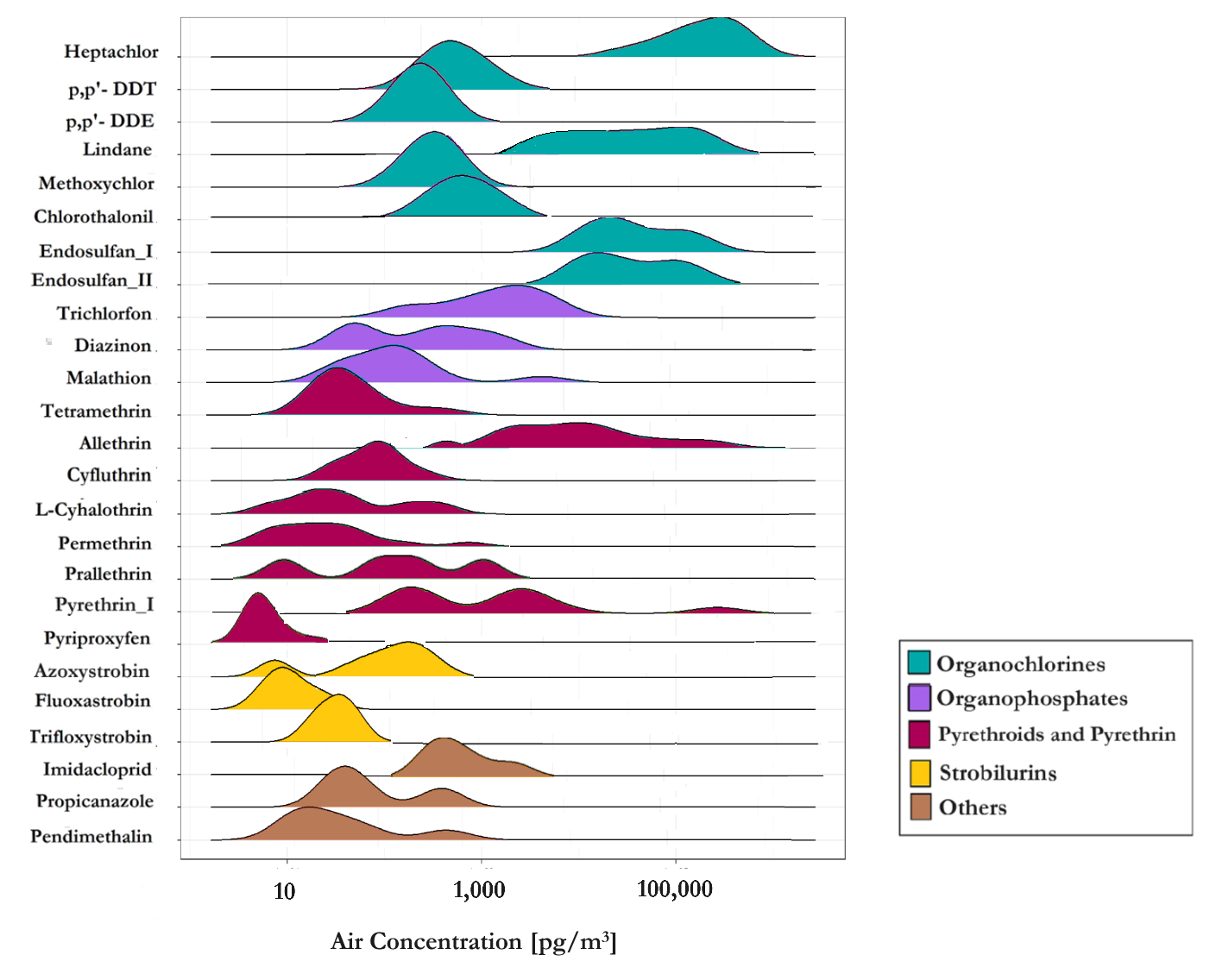


Figure S3. The total air concentrations of detected pesticides in pg/m^3^. Total air is the sum of the particle-phase and estimated gas-phase concentrations obtained using Equations 2 and 3 with values of Koa obtained from OPERA2.6.

**SI10: Literature Values**

Table S9. Summary of air concentrations (pg/m^3^) for detected pesticides reported in the literature (DL: detection limit). Color coding indicates detection frequencies (see below).

| **Reference** | Current study | Rudel et al., (6) | Tapia et al, (7)^*^ | | Bouvier et al., (8) | | Lewis et al., (9) | Tulve et al., (10) | | Audy et al., (11) | Lu et al., (12)^*^ | | Whyatt et al., (13) ^*^ | Rudel et al, (14)* |
| --- | --- | --- | --- | --- | --- | --- | --- | --- | --- | --- | --- | --- | --- | --- |
| **Country** | 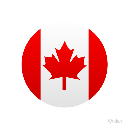 | 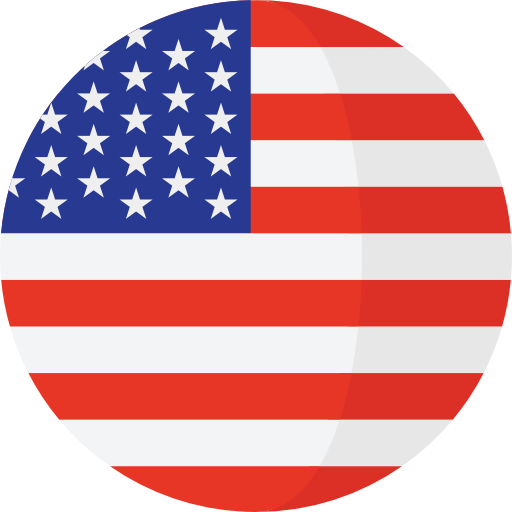 | 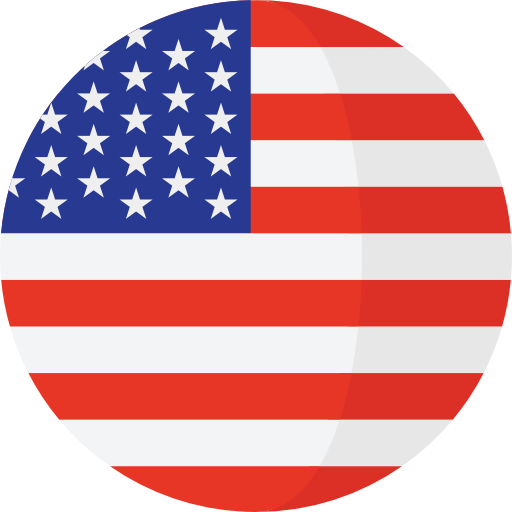 | | 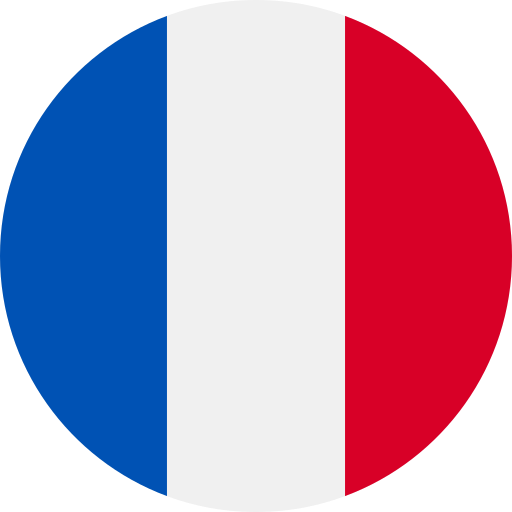 | | 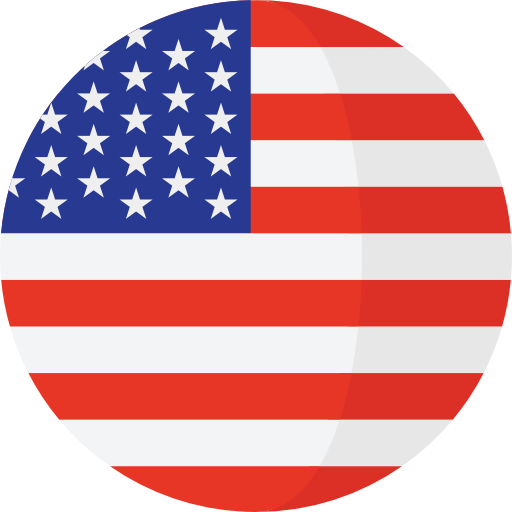 | 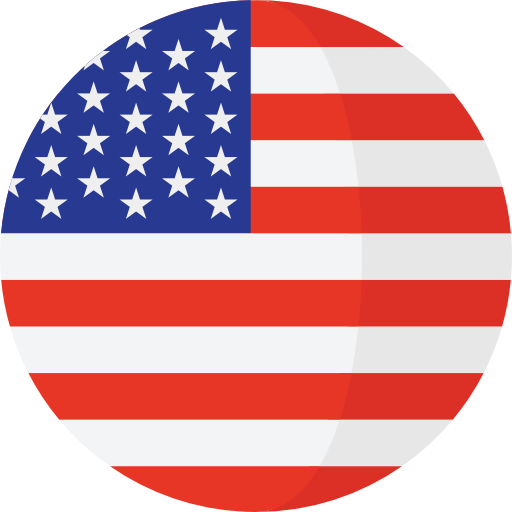 | | 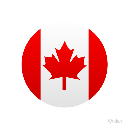 | | 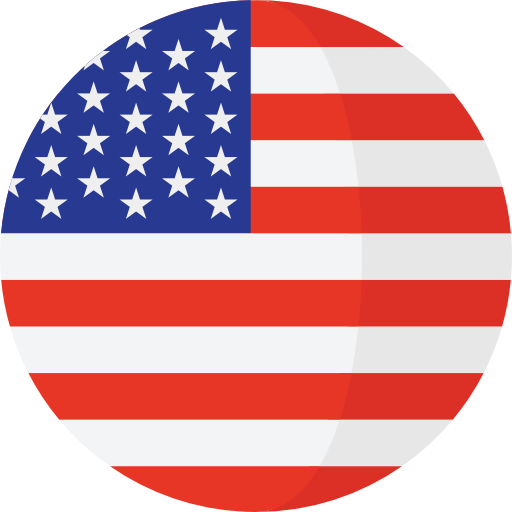 | 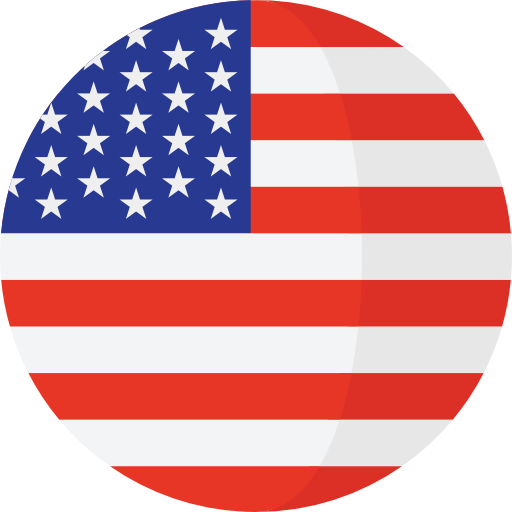 | 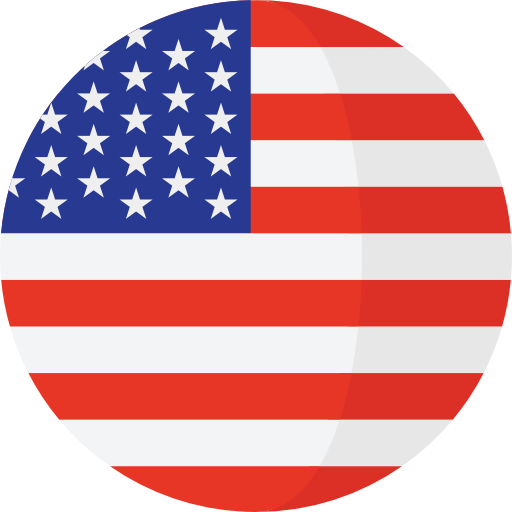 |
| **Sampling Method** | Portable air cleaner-particle phase | Active air -total air | Active air- total air | | Active air- total air | | Active air- total air | Active air- total air | | Passive air-gas phase | Active air-gas phase | | Active air- total air | Active air-total air |
| **Sample Size** | 46 | 119 | 25 | | 19 | | 47 | 9 | | 34 | 20 | | 102 | 50 |
| **Concentration (pg/m^3^)** | | | | | | | | | | | | | | |
| **Pesticides** |  |  | |  | |  |  | |  |  | |  |  |  |
| Heptachlor | **<DL-2,600** | **<DL- 71,000** | |  | |  | **Maximum of 200,000** | |  |  | |  |  | **<DL-5,000** |
| p,p’-DDT | **<DL-1,400** | **<DL-30,000** | |  | | **<DL-900** |  | |  | **<DL - 563** | |  |  | **<DL-1,700** |
| p,p’- DDE | **<DL-240** | **<DL-5,100** | |  | |  |  | |  | **24.7 - 776** | |  |  | **<DL-1,200** |
| Lindane | **<DL-990** | **<DL-110,000** | |  | | **<DL-72,200** |  | |  | **43.7 – 2,150** | |  |  |  |
| Methoxychlor | **<DL-1,200** |  | |  | |  |  | |  |  | |  |  |  |
| Chlorothalonil | **<DL-1,200** | **<DL-36,000** | |  | |  |  | |  |  | |  |  | **<DL-27,000** |
| Endosulfan-I | **<DL-1,100** |  | |  | | **<DL-13,500** |  | |  |  | |  |  |  |
| Endosulfan-II | **<DL-760** |  | |  | | **<DL-1,100** |  | |  |  | |  |  |  |
| Trichlorfon | **<DL-3,600** |  | |  | |  |  | |  |  | |  |  |  |
| Diazinon | **<DL-760** | **<DL-550,000** | | **<DL- 2,300** | | **<DL- 12,000** |  | |  |  | | **<DL-2,300** | **400–641,000** | **<DL-31,000** |
| Malathion | **<DL-2,800** |  | | **<DL-8,800** | |  |  | |  |  | |  |  |  |

| **Reference** | Current study | Rudel et al., (6) | Tapia et al, (7)^*^ | Bouvier et al., (8) | Lewis et al., (9) | Tulve et al., (10) | Audy et al., (11) | Lu et al., (12)^*^ | Whyatt et al., (13) ^*^ | Rudel et al, (14)* |
| --- | --- | --- | --- | --- | --- | --- | --- | --- | --- | --- |
| **Country** | 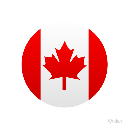 | 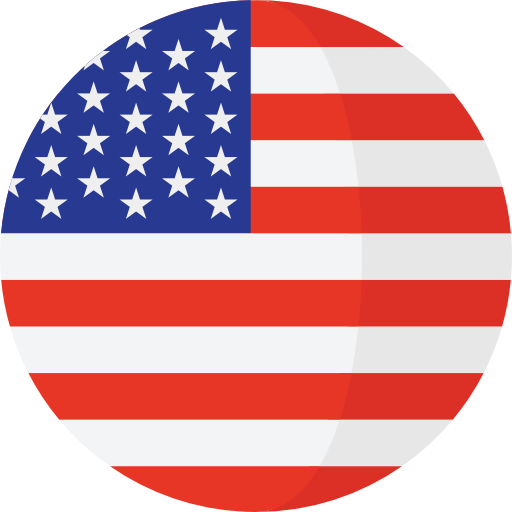 | 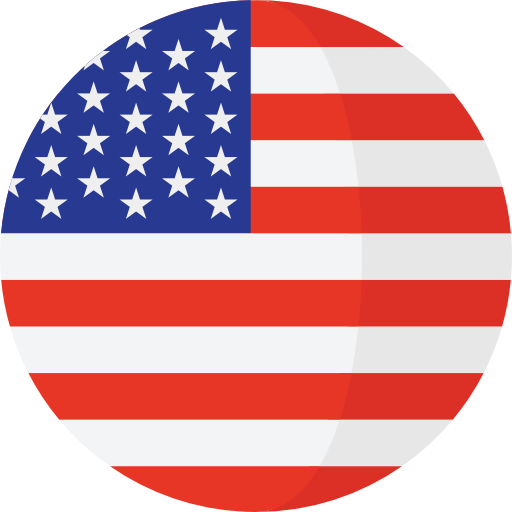 | 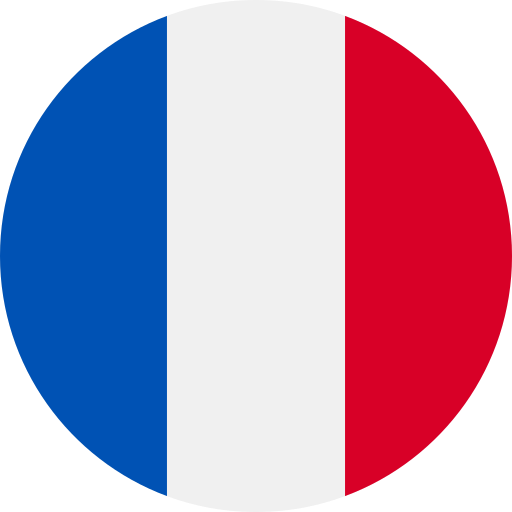 | 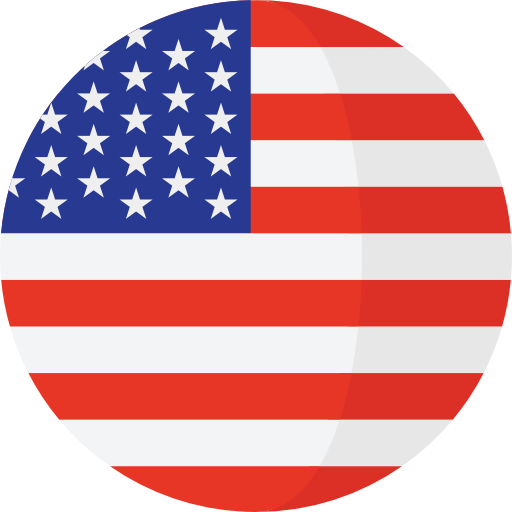 | 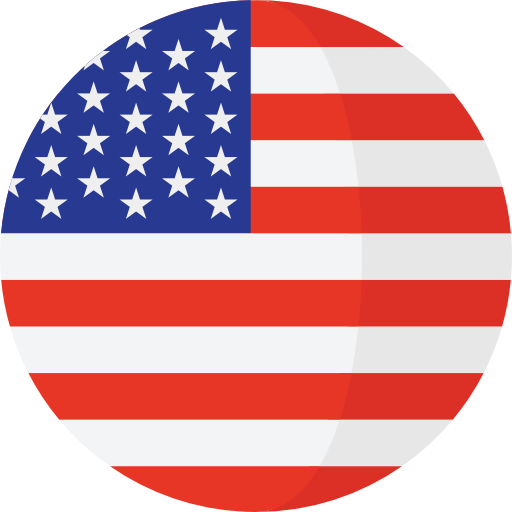 | 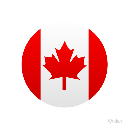 | 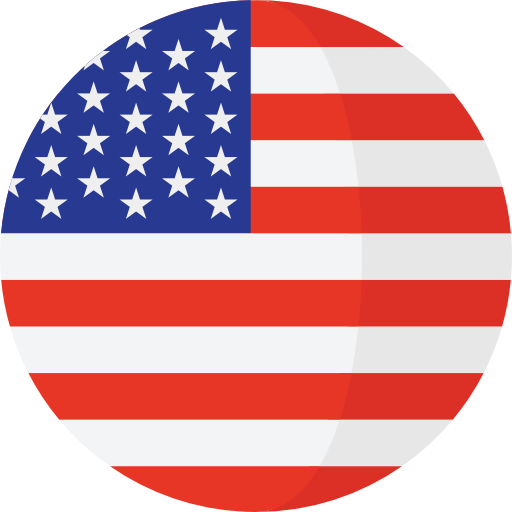 | 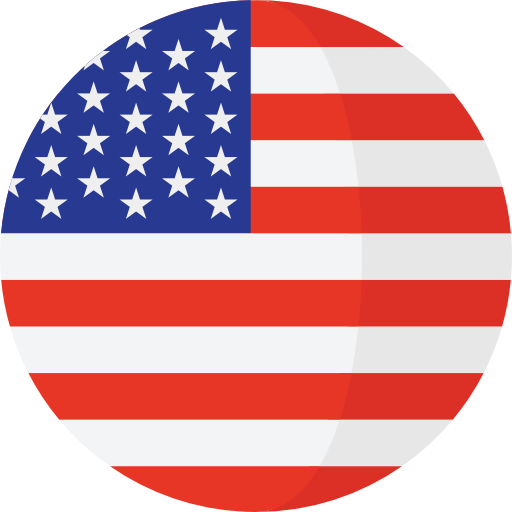 | 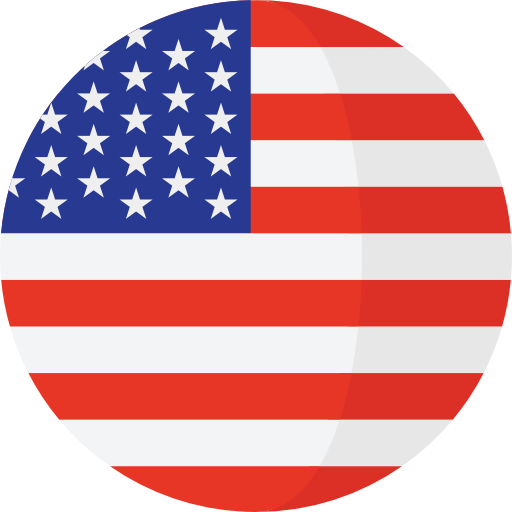 |
| **Sampling Method** | Portable air cleaner-particle phase | Active air -total air | Active air- total air | Active air- total air | Active air- total air | Active air- total air | Passive air-gas phase | Active air-gas phase | Active air- total air | Active air-total air |
| **Concentration (pg/m3)** | | | | | | | | | | |
| Tetramethrin | **<DL-5,000** |  |  |  |  | **<DL-63,000** |  |  |  |  |
| Allethrin | **<DL-16,000** |  |  |  |  | **<DL-74,000** |  | **<DL-3,760** |  |  |
| Cyfluthrin | **<DL-3,300** |  |  |  |  | **<DL- 5,500** |  |  |  |  |
| L-Cyhalothrin | **<DL- 6,000** |  |  |  |  |  |  | **<DL-9,180** |  |  |
| Permethrin | **<DL-14,000** | **<DL- 5,400** | **<DL- 1,000** |  |  | **<DL-130,000** |  | **<DL-3,030** | **<DL- 164,000** | **<DL-3,000** |
| Prallethrin | **<DL-380** |  |  |  |  |  |  |  |  |  |
| Pyrethrin I | **<DL-32,000** |  |  |  |  | **<DL-12,000** |  |  |  |  |
| Pyriproxyfen | **<DL-47** |  |  |  |  |  |  |  |  |  |
| Azoxystrobin | **<DL-1,100** |  |  |  |  |  |  |  |  |  |
| Fluoxastrobin | **<DL-94** |  |  |  |  |  |  |  |  |  |
| Trifloxystrobin | **<DL-120** |  |  |  |  |  |  |  |  |  |
| Pendimethalin | **<DL-4,400** |  |  |  |  |  |  |  |  |  |

| **Detection frequency %** | **0-29** | **30-49** | **50-79** | **80-100** |
| --- | --- | --- | --- | --- |
| *L* Low and middle-income families | | | | |

**SI11: Spearman Rank Correlation and Upset Plot**

Table S10. Spearman rank correlation (rho) for pesticides in 46 social housing MURBs.

|  | Heptachlor | p,p’-DDT | p,p’-DDE | Lindane | Methoxychlor | Chlorothalonil | Endosoulfan_I | Endosulfan_II | Trichlorfon | Diazinon | Malathion | Tetramethrin | Allethrin | Cyfluthrin | L-Cyhalothrin | Permethrin | Prallethrin | Pyrethrin I | Pyriproxyfen | Azoxystrobin | Fluoxastrobin | Trifloxystrobin | Imidacloprid | Propiconazole | Pendimethalin |
| --- | --- | --- | --- | --- | --- | --- | --- | --- | --- | --- | --- | --- | --- | --- | --- | --- | --- | --- | --- | --- | --- | --- | --- | --- | --- |
| Heptachlor |  |  |  |  |  |  |  |  |  |  |  |  |  |  |  |  |  |  |  |  |  |  |  |  |  |
| p,p’-DDT |  |  |  |  |  |  |  |  |  |  |  |  |  |  |  |  |  |  |  |  |  |  |  |  |  |
| p,p’-DDE |  |  |  |  |  |  |  |  |  |  |  |  |  |  |  |  |  |  |  |  |  |  |  |  |  |
| Lindane |  |  |  |  |  |  |  |  |  |  |  |  |  |  |  |  |  |  |  |  |  |  |  |  |  |
| Methoxychlor |  |  |  |  |  |  |  |  |  |  |  |  |  |  |  |  |  |  |  |  |  |  |  |  |  |
| Chlorothalonil |  |  |  |  |  |  |  |  |  |  |  |  |  |  |  |  |  |  |  |  |  |  |  |  |  |
| Endosoulfan_I |  |  |  |  |  |  |  |  |  |  |  |  |  |  |  |  |  |  |  |  |  |  |  |  |  |
| Endosulfan_II |  |  |  |  |  |  |  |  |  |  |  |  |  |  |  |  |  |  |  |  |  |  |  |  |  |
| Trichlorfon |  |  |  |  |  |  |  |  |  |  |  |  |  |  |  |  |  |  |  |  |  |  |  |  |  |
| Diazinon |  |  |  |  |  |  |  |  |  |  |  |  |  |  |  |  |  |  |  |  |  |  |  |  |  |
| Malathion |  |  |  |  |  |  |  |  |  |  |  |  |  |  |  |  |  |  |  |  |  |  |  |  |  |
| Tetramethrin |  |  |  |  |  |  |  |  |  |  |  |  |  |  |  |  |  |  |  |  |  |  |  |  |  |
| Allethrin |  |  |  |  |  |  |  |  |  |  |  |  |  |  |  |  |  |  |  |  |  |  |  |  |  |
| Cyfluthrin |  |  |  |  |  |  |  |  |  |  |  |  |  |  |  |  |  |  |  |  |  |  |  |  |  |
| L-Cyhalothrin |  |  |  |  |  |  |  |  |  |  |  |  |  |  |  |  |  |  |  |  |  |  |  |  |  |
| Permethrin |  |  |  |  |  |  |  |  |  |  |  |  |  |  |  |  |  |  |  |  |  |  |  |  |  |
| Prallethrin |  |  |  |  |  |  |  |  |  |  |  |  |  |  |  |  |  |  |  |  |  |  |  |  |  |
| Pyrethrin I |  |  |  |  |  |  |  |  |  |  |  |  |  |  |  |  |  |  |  |  |  |  |  |  |  |
| Pyriproxyfen |  |  |  |  |  |  |  |  |  |  |  |  |  |  |  |  |  |  |  |  |  |  |  |  |  |
| Azoxystrobin |  |  |  |  |  |  |  |  |  |  |  |  |  |  |  |  |  |  |  |  |  |  |  |  |  |
| Fluoxastrobin |  |  |  |  |  |  |  |  |  |  |  |  |  |  |  |  |  |  |  |  |  |  |  |  |  |
| Trifloxystrobin |  |  |  |  |  |  |  |  |  |  |  |  |  |  |  |  |  |  |  |  |  |  |  |  |  |
| Imidacloprid |  |  |  |  |  |  |  |  |  |  |  |  |  |  |  |  |  |  |  |  |  |  |  |  |  |
| Propiconazole |  |  |  |  |  |  |  |  |  |  |  |  |  |  |  |  |  |  |  |  |  |  |  |  |  |
| Pendimethalin |  |  |  |  |  |  |  |  |  |  |  |  |  |  |  |  |  |  |  |  |  |  |  |  |  |
|  |  |  |  |  |  |  |  |  |  |  |  |  |  |  |  |  |  |  |  |  |  |  |  |  |  |
|  |  | Correlation significant at level 0.01 | | | | | |  |  |  |  |  |  |  |  |  |  |  |  |  |  |  |  |  |  |
|  |  | Correlation significant at level 0.05 | | | | | | |  |  |  |  |  |  |  |  |  |  |  |  |  |  |  |  |  |
|  |  |  | | | | | |  |  |  |  |  |  |  |  |  |  |  |  |  |  |  |  |  |  |

**
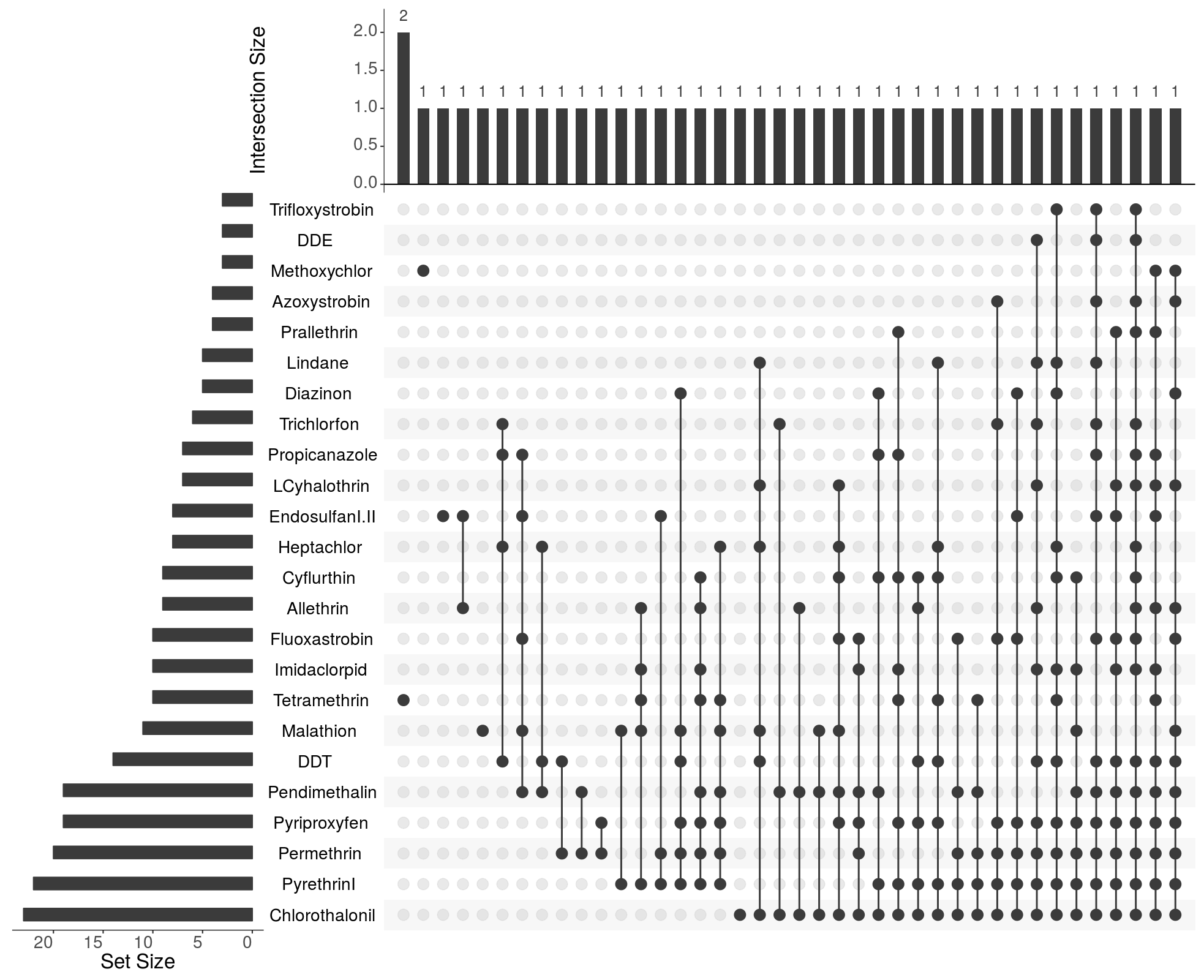
**

Figure S4. Upset plot showing the co-occurrences of pesticides within 46 units. Units with no detected pesticides were not included in this graph.

**SI12: Influence of Tobacco Smoking**

Table S11. Mann-Whitney-Wilcoxon p values for pesticides with DF>60% in smoker versus non-smoker units. Values <MDL for pesticides with DF>60% were replaced by ½ MDL.

| **Smoker/**  **Non-smoker** | **Pesticides** | | | | |  |
| --- | --- | --- | --- | --- | --- | --- |
|  | **Chlorothalonil** | **Pendimethalin** | **Permethrin** | **Pyrethrin I** | **Pyriproxyfen** | |
| p-values | 2.7 x 10^-5^ | 0.002 | 0.03 | 0.0022 | 0.044 | |


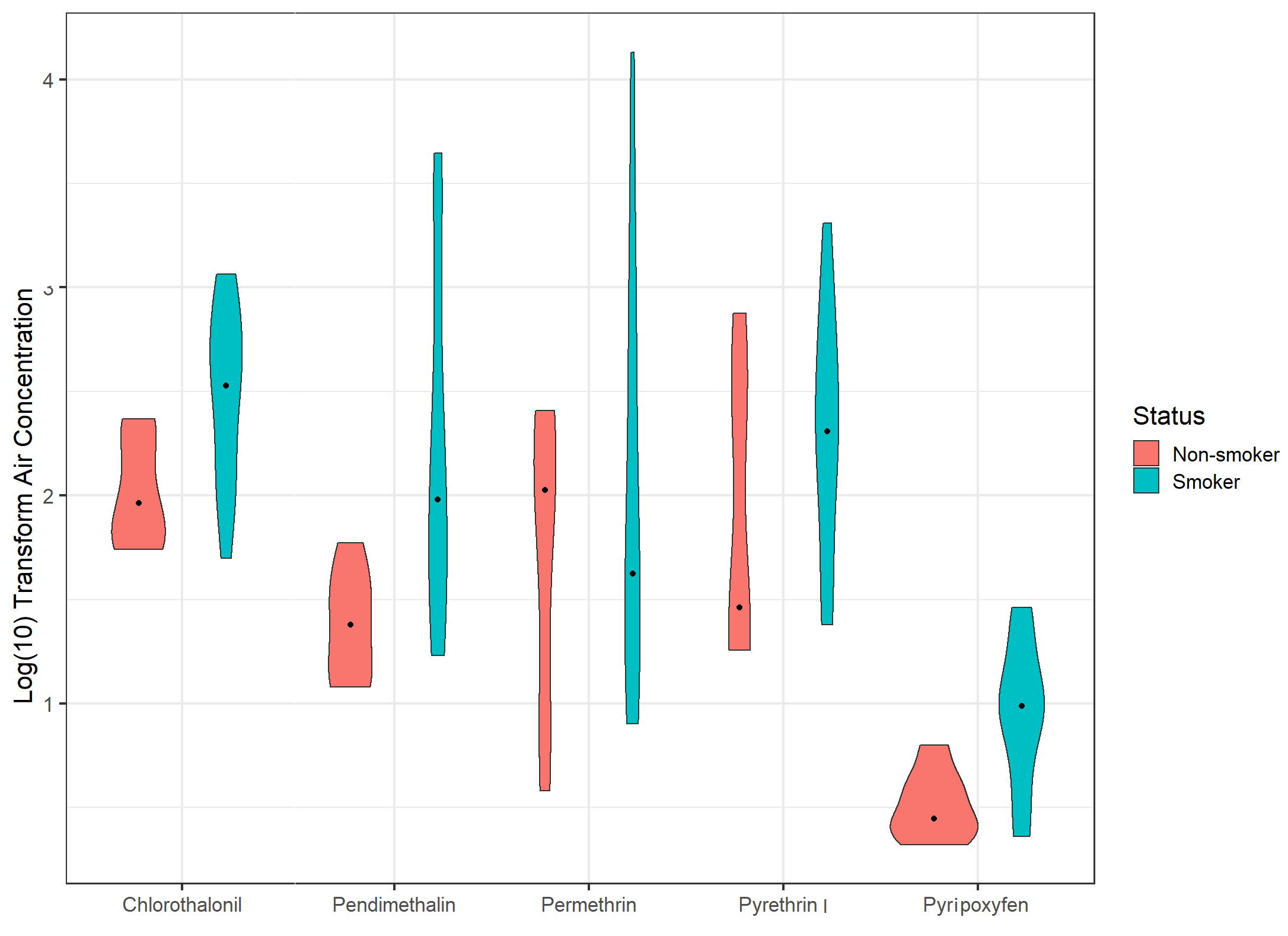


Figure S5. A violin plot comparing concentrations of pesticides with DF>60% in units with evidence of tobacco smoking vs units with no evidence of tobacco smoking. The concentrations are log-transformed. The median values are shown with a “black dot”.

**References**

1. Wan Y, Diamond ML, Siegel JA. Quantitative filter forensics for semivolatile organic compounds in social housing apartments. Indoor Air. 2022;32(2):e12994.

2. Harner T, Bidleman TF. Octanol-air partition coefficient for describing particle/gas partitioning of aromatic compounds in urban air. Environ Sci Technol. 1998;32(10):1494–502.

3. United States Environmental Protection Agency. CompTox Chemicals Dashboard. 2023. Available from: https://comptox.epa.gov/dashboard/

4. Navaranjan G, Jantunen LM, Diamond ML, Harris SA, Bernstein S, Scott JA, et al. Early Life Exposure to Tris(2-butoxyethyl) Phosphate (TBOEP) Is Related to the Development of Childhood Asthma. Environ Sci Technol Lett. 2021;8(7):531–7.

5. US EPA. Estimation Programs Interface Suite^TM^ for Microsoft® Windows, v 4.11. United States Environmental Protection Agency. Washington, DC: USA; 2023.

6. Rudel RA, Camann DE, Spengler JD, Korn LR, Brody JG. Phthalates, Alkylphenols, Pesticides, Polybrominated Diphenyl Ethers, and Other Endocrine-Disrupting Compounds in Indoor Air and Dust. Environ Sci Technol. 2003;37(20):4543–53.

7. Tapia, B., Bortoni, P., Escobedo, E., Camann, D., Heilbrun, L. P., Whyatt, R. M., & Miller CS. A Comparitive Study of Pesticide Use in Homes of Pregnant Women Living at the Texas-Mexico Border and in New York City. Texas Public Heal J. 2012;64(3):18–23.

8. Bouvier G, Blanchard O, Momas I, Seta N. Pesticide exposure of non-occupationally exposed subjects compared to some occupational exposure: A French pilot study. Sci Total Environ. 2006;366(1):74–91.

9. Lewis RG, Fortmann RC, Camann DE. Evaluation of methods for monitoring the potential exposure of small children to pesticides in the residential environment. Arch Environ Contam Toxicol. 1994;26(1):37–46.

10. Tulve NS, Egeghy PP, Fortmann RC, Whitaker DA, Nishioka MG, Naeher LP, et al. Multimedia measurements and activity patterns in an observational pilot study of nine young children. J Expo Sci Environ Epidemiol. 2008;18(1):31–44.

11. Audy O, Melymuk L, Venier M, Vojta S, Becanova J, Romanak K, et al. PCBs and organochlorine pesticides in indoor environments - A comparison of indoor contamination in Canada and Czech Republic. Chemosphere. 2018;206:622–31. Available from: https://doi.org/10.1016/j.chemosphere.2018.05.016

12. Lu C, Adamkiewicz G, Attfield KR, Kapp M, Spengler JD, Tao L, et al. Household pesticide contamination from indoor pest control applications in urban low-income public housing dwellings: A community-based participatory research. Environ Sci Technol. 2013;47(4):2018–25.

13. Whyatt RM, Garfinkel R, Hoepner LA, Holmes D, Borjas M, Williams MK, et al. Within- and Between-Home Variability in Indoor-Air Insecticide Levels during Pregnancy among an Inner-City Cohort from New York City. Environ Health Perspect. 2007;115(3):383–9.

14. Rudel RA, Dodson RE, Perovich LJ, Morello-Frosch R, Camann DE, Zuniga MM, et al. Semivolatile Endocrine-Disrupting Compounds in Paired Indoor and Outdoor Air in Two Northern California Communities. Environ Sci Technol. 2010;44(17):6583–90.
